# Targeted delivery of a PD-1-blocking scFv by CD133-specific CAR-T cells using nonviral Sleeping Beauty transposition shows enhanced antitumour efficacy for advanced hepatocellular carcinoma

**DOI:** 10.1186/s12916-023-03016-0

**Published:** 2023-08-28

**Authors:** Chaopin Yang, Jinqi You, Qiuzhong Pan, Yan Tang, Liming Cai, Yue Huang, Jiamei Gu, Yizhi Wang, Xinyi Yang, Yufei Du, Dijun Ouyang, Hao Chen, Haoran Zhong, Yongqiang Li, Jieying Yang, Yulong Han, Fengze Sun, Yuanyuan Chen, Qijing Wang, Desheng Weng, Zhongqiu Liu, Tong Xiang, Jianchuan Xia

**Affiliations:** 1 Department of Experimental Research, State Key Laboratory of Oncology in South China, Collaborative Innovation Center for Cancer Medicine, Sun Yat-Sen University Cancer Center, Guangzhou, Guangdong 510060 People’s Republic of China; 2 Department of Biotherapy, Sun Yat-Sen University Cancer Center, Guangzhou, Guangdong 510060 People’s Republic of China; 3 International Institute for Translational Chinese Medicine, Guangzhou University of Chinese Medicine, Guangzhou, Guangdong 510060 People’s Republic of China; 4 Department of Molecular Diagnostics, Sun Yat-Sen University, Cancer Center, Guangzhou, Guangdong 510060 People’s Republic of China; 5 Department of Thoracic Surgery, Sun Yat-Sen University Cancer Center, Guangzhou, Guangdong 510060 People’s Republic of China

**Keywords:** Sleeping Beauty system, Minicircle, CAR-T, CD133, PD-1, Hepatocellular carcinoma

## Abstract

**Background:**

CD133 is considered a marker for cancer stem cells (CSCs) in several types of tumours, including hepatocellular carcinoma (HCC). Chimeric antigen receptor-specific T (CAR-T) cells targeting CD133-positive CSCs have emerged as a tool for the clinical treatment of HCC, but immunogenicity, the high cost of clinical-grade recombinant viral vectors and potential insertional mutagenesis limit their clinical application.

**Methods:**

CD133-specific CAR-T cells secreting PD-1 blocking scFv (CD133 CAR-T and PD-1 s cells) were constructed using a sleeping beauty transposon system from minicircle technology, and the antitumour efficacy of CD133 CAR-T and PD-1 s cells was analysed in vitro and in vivo.

**Results:**

A univariate analysis showed that CD133 expression in male patients at the late stage (II and III) was significantly associated with worse progression-free survival (PFS) (*P* = 0.0057) and overall survival (OS) (*P* = 0.015), and a multivariate analysis showed a trend toward worse OS (*P* = 0.041). Male patients with advanced HCC exhibited an approximately 20-fold higher PD-L1 combined positive score (CPS) compared with those with HCC at an early stage. We successfully generated CD133 CAR-T and PD-1 s cells that could secrete PD-1 blocking scFv based on a sleeping beauty system involving minicircle vectors. CD133 CAR-T and PD-1 s cells exhibited significant antitumour activity against HCC in vitro and in xenograft mouse models. Thus, CD133 CAR-T and PD-1 s cells may be a therapeutically tractable strategy for targeting CD133-positive CSCs in male patients with advanced HCC.

**Conclusions:**

Our study provides a nonviral strategy for constructing CAR-T cells that could also secrete checkpoint blockade inhibitors based on a Sleeping Beauty system from minicircle vectors and revealed a potential benefit of this strategy for male patients with advanced HCC and high CD133 expression (median immunohistochemistry score > 2.284).

**Supplementary Information:**

The online version contains supplementary material available at 10.1186/s12916-023-03016-0.

## Background

Hepatocellular carcinoma (HCC) is the most common primary liver malignancy and the third leading cause of cancer-related death worldwide [1]. Despite the evaluation of many chemotherapies and targeted therapeutic agents, the only proven treatments for advanced disease are sorafenib, regorafenib and lenvatinib, and the overall survival benefits are modest [2]. Recently, two programmed cell death protein 1 (PD-1) inhibitors, pembrolizumab [3] and nivolumab [4], were approved for HCC treatment in patients who experienced progression after taking sorafenib, but their response rates (approximately 20%) were also unsatisfactory [5]. Thus, a new treatment strategy that improves clinical benefits for HCC is urgently needed.

Recurrence and metastasis are frequent and expected events after systemic therapy in patients treated with current treatments for advanced HCC. Emerging evidence suggests that most systemic therapies can only diminish the primary tumour load because cancer stem cells (CSCs) possess intrinsic mechanisms to resist traditional or novel therapy, and resting CSCs are responsible for tumour initiation, recurrence and metastasis [6, 7]. Along with the presence of CD133-positive HCC stem cell-like cells [8], a growing number of studies indicate that CD133 expression is correlated with higher stage tumours and poor prognosis in HCC patients [9, 10], suggesting a promising strategy of using CD133 as a target for HCC treatment. Chimeric antigen receptor-specific T (CAR-T) cells targeting CD133-positive CSCs have emerged as a tool for the clinical treatment of HCC (NCT02541370).

CAR-T cell-based immune therapy for cancers is based on stably integrating a transgene into the host genome so that infused genetically modified progeny can provide long-term therapeutic effects. To date, most CAR-T cell therapies are produced via traditional viral vector systems that have been extensively and safely used to insert transgenes into T cells, but immunogenicity, the high cost of clinical-grade recombinant viral vectors and the potential for insertional mutagenesis limit their clinical application [11, 12]. Nonviral gene transfer systems are attractive alternatives to current methods for producing CAR-T cells. The most common alternatives to viral vectors are transposons. A variety of transposon-based systems have been reported for CAR-T cell production, and these systems provide lower immunogenicity, enhanced safety profiles and reduced costs for GMP manufacture of CAR-T cells. Of the available technologies now in clinical trials, the sleeping beauty (SB) transposon system has experienced the most rapid development from inception to application in humans [13]. Minicircles (MCs) are supercoiled DNA vectors that constitute a novel alternative to plasmids as sources of SB transposases and transposons. Compared to conventional plasmids, MCs lack bacterial backbone sequences and antibiotic resistance genes, resulting in more stable and higher levels of transient gene expression when transfected into mammalian cells [14, 15].

In this paper, we describe a protocol for generating CD133-specific CAR-T cells secreting PD-1 blocking single-chain variable fragment (scFv) targeting HCC cells using an SB-based nonviral genetic modification system involving MC transposon donor vectors to simplify the current CAR-T cell production protocol. We aimed to use our CD133-specific CAR-T cell platform to create a single therapy against advanced HCC in which PD-1 blocking scFv would be delivered locally to the site of the tumour to minimize toxicity.

## Methods

### Patients

For immunohistochemical (IHC) staining, 67 hepatocellular carcinoma (HCC) patient samples, consisting of adjacent non‐cancerous tissues and HCC tumour tissues, were obtained from patients who underwent primary HCC resection at Sun Yat-sen University Cancer Centre between 2005 and 2008. These patients did not receive any preoperative chemotherapy or radiotherapy. Additionally, the study included 26 cases of lung metastasis, collected from patients who underwent lung metastasis resection at the same centre between 2003 and 2021.

All human samples used in the study were archived materials obtained with informed consent from the patients. The use of these samples was approved by the Institutional Review Board of SYSUCC (Approval No. GZR2019-287).

### Plasmid construction

The plasmids used in this study were custom-cloned by vectorbuilder.com. The following SB transposon gene expression vectors were used:

1. pSB-EF1alpha-antiCD133 CD8 4-1BB CD3z-P2A-PD-1 blocking scFv-WPRE-BGH polyA

The anti-CD133 scFv derived from HW350341.1 in a previous study [16] is expressed under the control of the EF1 alpha promoter. To assess the gene transfer efficiency and the secretion of PD-1 blocking scFv, we inserted a c-Myc-tag (GAGCAGAAACTCATCTCTGAAGAGGATCTG) between the CD133 scFv and CD8TM and at the end of PD-1 blocking scFv, respectively. The CD133 CAR and PD-1 blocking scFv were linked by a P2A peptide sequence. To increase gene expression, we placed a woodchuck hepatitis virus posttranscriptional regulatory element (WPRE) between the stop codon and the bovine growth hormone (BGH) polyA signal. The inverted terminal repeats (ITRs) with specific binding to SB transposase identified in a previous study [17] were placed before EF-1α and at the end of BGH polyA. The sequence of pSB-EF1alpha-antiCD133 CD8 4-1BB CD3z-P2A-PD-1 blocking scFv-WPRE-BGH polyA was also attached as in Additional file 1.

(2) pSB-EF1alpha-antiCD133 CD8 4-1BB CD3z -WPRE-BGH polyA

To compare the efficiency of anti-CD133 CAR-T cells and anti-CD133 CAR-T cells secreting PD-1 blocking scFv, we also constructed anti-CD133 CAR-T cells with the same structure described in (1) without the PD-1 blocking scFv.

(3) pSB-CMV promoter-SV40 intron-SB100 × -SV40 poly(A) signal

pCMV(CAT)T7-SB100 was purchased from Addgene (catalogue number 34879). We used homologous recombination CMV promoter-SV40 intron-SB100 × -SV40 poly (A) into MCS sites in a Parental Minicircle Cloning Vector (pMC. BESPX-MCS2, catalogue number MN100B-1, System Biosciences).

(4) pMC.BESPX-MCS2

pMC.BESPX-MCS2 is an empty parental minicircle cloning vector (catalogue number MN100B-1, System Biosciences), which was used to produce minicircles from transposons and transposases.

(5) Minicircle Production Strain

ZYCY10P3S2T *E. coli* (catalogue number MN900A-1, System Biosciences) was the competent cell used for minicircle production.

### Minicircle DNA plasmid production

#### Inoculation

We first cloned the SB transposon *pSB-EF1alpha-antiCD133 CD8 4-1BB CD3z-P2A-PD-1 blocking scFv-WPRE-BGH polyA*, *pSB-EF1alpha-antiCD133 CD8 4-1BB CD3z-WPRE-BGH polyA* and SB100 × transposase into the MCS sites of the parental minicircle cloning vector (pMC.BESPX-MCS2), added 200 ng of the plasmid into one vial of ZYCY10P3S2T *E. coli* by gently rotating the pipette tip through the cells while dispensing, incubated on ice for 30 min, heat-shocked the cells for 30 s in a 42 °C water bath, and then placed the cells on ice for 2 min. We added 0.2 ml of room temperature S.O.C. medium, transferred the sample to a bacterial culture tube and incubated the cells with shaking at 250 rpm (37 °C) for 60 min. Prewarmed LB culture plates for kanamycin selection (50 µg/ml kanamycin) were then spread with 50–200 μl of bacterial. The cells were incubated overnight at 37 °C. Then, 3–5 colonies were picked and grown in 2 ml of LB containing 50 μg/ml kanamycin. The cells were grown overnight, the plasmid was extracted by miniprep, and the minicircle parental plasmid was evaluated.

A single colony was picked into 2 ml of LB containing 50 μg/ml kanamycin and then incubated at 30 °C with shaking at 250 rpm for 4–6 h. Then, 0.5–1 ml to 200 ml of 1 × growth medium was added. The plates were placed back into an incubator at 30 °C with shaking at 250 rpm overnight.

#### Induction

After overnight growth, the pH and OD600 of the culture medium were measured. The target pH was approximately 7, and the target OD600 was 4–6. Then, 400 ml of the overnight culture was combined with 400 ml of 1 × induction medium (400 ml of fresh LB medium, 16 ml of 1 N sodium hydroxide and 0.4 ml of 20% L-arabinose). The mixture was incubated again at 32 °C and shaken at 250 rpm for 5 h. The total induction time was 5 h. One millilitre of the bacterial culture was used to perform a miniprep, followed by restriction digest analysis to check the quality of the minicircle plasmid. Bacteria were pelleted at 4 °C, and the pellet was stored at − 80 °C.

An endotoxin-free plasmid extraction kit (catalogue number DP117, TIANGEN) was used to extract the plasmid from the bacterial pellet. Then, the plasmid was cut using the restriction site SalI to assess whether the minicircle was successfully induced. The minicircle size was smaller than that of the parent plasmid, and there was no parent plasmid contamination.

#### T cell isolation and CAR-T cell electrotransfection

PBMCs were obtained from volunteers, and buffy coats were obtained from MD Pacific Cell Separation Media (MD Pacific, Tianjin, China). After erythrocyte lysis (Beyotime, China), T cells were isolated using the Pan T Cell Isolation Kit (Miltenyi, Bergisch Gladbach, Germany). Then, T cells were activated by anti-CD3/CD28 bead stimulation (Stemcell, USA) overnight. Transfection of transposon and transposase vectors into 1 × 10^7^ T cells was performed on day 2 with a 4D-Nucleofector according to the manufacturer’s instructions (Lonza, Cologne, Germany). Electroporation was performed with the Amaxa™ P3 Primary Cell 4D-Nucleofector™ X Kit L (24 RCT). Briefly, 1 × 10^7^ cells were suspended in 100 μL of nucleofection buffer containing the indicated amounts of transposon and transposase vector DNA. Program E0-115 was applied, and cells were immediately supplied with 3 mL of medium (X-vivo) with 1000 IU/ml recombinant human interleukin (IL)-2, IL-7, and IL-15 culture for 2 ~ 3 days. Forty-eight to seventy-two hours after electroporation, the cells were resuspended in 2 mL fresh medium (X-vivo) with 400 IU/ml recombinant human interleukin (IL)-2 (Four Rings Biopharmaceutical, Beijing, China) and expanded with CD133 recombinant protein antigen (R andD Systems, USA) and CD28 for 2–3 days. The CAR-T-positive cells were evaluated by flow cytometry through the c-Myc-tag, enriched using biotin-conjugated c-Myc-tag (CST, USA) and anti-biotin beads (Miltenyi, Germany) and expanded for up to 21 ~ 28 days. The concentration of IL-2 was reduced to 40 IU/ml 3–4 days prior to functional analysis.

### Lentiviral engineering of tumour cell lines and ex vivo T cell transduction

Lentiviral stocks were generated by transfection of 293 T cells with the luciferase, blasticidin and puromycin plasmids pMD2.G and pSPAX (Addgene). For lentiviral gene transfer into tumour cell lines, 20 μl lentivirus was added to tumour cells. Two days after infection, the positive tumour cells were selected using blasticidin. For lentiviral gene transfer into T cells, an MOI of 10 was used to infect primary T cells from volunteers. After sorting using puromycin, PD + 1 T cells were assessed by flow cytometry.

### Immunohistochemistry

The mounting slides were incubated at 65 °C for 2.5 h, hydrated, repaired in 3% H_2_O_2_, blocked in goat serum, incubated with the primary antibody against CD133 (Invitrogen, catalogue number PA5-82,184, 1:250 dilution), and then incubated with the secondary antibody (Dako, Agilent). The slides were scanned through a digital pathology section scanner (KFBIO, Ningbo, China) to obtain a whole scan. The amplified view was photographed under a microscope (Nikon Eclipse Ni-U).

Semiquantitative scoring was performed as follows: 0 for staining in ≤ 1% of cancer cells, 1 for staining in 2–25% of cancer cells, 2 for staining in 26–50% of cancer cells, 3 for staining in 51–75% of cancer cells, and 4 for staining in ≥ 75% of cancer cells. Staining intensity was scored as follows: 0, no staining; 1, weak staining; 2, moderate staining; and 3, strong staining.

Tumour programmed death ligand 1 (PD-L1) expression was assessed using a PD-L1 immunohistochemistry (IHC) SP142 antibody clone (Abcam, 228,462) and combined positive score (CPS). CPS, which is a highly reproducible PD-L1 scoring method for multiple tumour types [18, 19], is obtained by summing the number of PD-L1-stained cells (tumour cells, lymphocytes, macrophages), dividing the result by the total number of viable tumour cells, and multiplied by 100 and CPS/entire tumour area based on the following formula:

$$CPS=\frac{NO.PD-L1\,stained\,cells\,(tumor\,cells,\,lymphocytes,\,macrophages)}{\begin{array}{c}\begin{array}{c}Total\,No.of\\ \end{array}\,viable\\ tumor\,cells\end{array}}\times\,100 \times\,(\mathrm{CPS\,of\,entire\,tumour\,area})$$

To assess whether the CAR-T cells infiltrated solid tumours, a CD3 antibody (catalogue number ZM-0417–6.0, OriGene, working solution) was used to assess whether the T cells had infiltrated into solid tumours.

### Flow cytometry

Flow cytometry was used to determine the transduction efficiency of transduced cells following staining with PE-conjugated c-Myc-tag (CST, clone 71D10), 7AAD, and APC-Cy7-conjugated rabbit anti-human CD3 (BD Biosciences, clone SK7, APC-Cy7). Data from cells were collected using a Beckman Coulter Gallios flow cytometer and analysed using CytExpert (Beckman). For analysis of CAR-T cells in the in vitro experiment, the following antibodies and reagents were used for flow cytometry to distinguish naïve T, effector T, central memory T, and effector memory T cells: APC-Cy7 anti-human CD3, PE anti c-Myc-tag (CST, clone, 71D10), APC-CD8 (BD, clone, HIT8α), FITC-CD45RA (BD, clone, L48), and PECy7-CCR7 (BD, clone, 3D12). The following antibodies were used to evaluate the frequencies of Tregs among CD4 T cells in the in vitro experiment: APC-Cy7 anti-human CD4 (BD, clone, RPA-T4), APC anti-human CD25(BD, clone, M-A251) and FITC anti-human Foxp3 (BioLegend, clone, 206D).

To evaluate the incubation of CAR-T and Hep3B cells, APC-Cy7-CD3 and PE-CD133 (Miltenyi, clone, REAL803) were used to distinguish CAR-T and Hep3B cells. PD-1-APC (BD, clone, MIH4) was used to evaluate PD-1 expression. To evaluate the PD-1 blocking scFv bystander effect, c-Myc-tagged PE was used to evaluate the supernatant of CD133 CAR-T and PD-1 s cells incubated with PD-1 + T cells, as the PE-conjugated anti-c-Myc-tag antibody can bind to PD-1 blocking scFv.

To evaluate CD107a expression, CAR-T cells incubated with Hep-3B, the CD107a (BD, clone, H4A3) antibody and a leukocyte activation cocktail with BD GolgiPlugTM (BD) and monensin (BioLegend) were added to the medium before analysis for 4 h.

To evaluate whether the PD-1 scFv could restore PD-1 + T cell function, FVS780 (BD, live/dead cells) was used to exclude dead cells. After washing with PBS, the cells were stained with CD3 PE (BD, clone, HIT3α). Intracellular staining for Granzyme B BV421 (BD, clone, GB11) and TNFα APC (BD, clone, MAb11) was finally performed according to the manufacturer’s protocol (BD Cytofix/Cytoperm™ Fixation/Permeabilization Solution Kit, No. 554714).

To evaluate T cells that infiltrated the tumour environment, the tumour was minced with a razor blade as fine as possible; subsequently, it was digested with collagenase I and IV (Yeason: #40507ES60 and #40510ES60), dispase II (Yeason: #40104ES80) and DNase I (Yeason: #10607ES15) in 1640 medium for 1 h to produce singe cells. The red blood cells were lysed and then subjected to CD3 APC (BD, clone, UCHT1) and live/dead FVS780 staining.

### Tumour killing assays

Continuous cytotoxicity assays involving CAR T cells were performed using an xCELLigence Real-Time Cell Analyzer-Multiple Plate system (Agilent Technologies). In brief, 11,000 Hep-3B cells were added to each well of a 16-well plate (Agilent, #20220922). After approximately 24 h, corresponding CAR-positive cells at different effector to target (E:T) ratios were added. The cell index values, which represent changes in the electrical impedance and reflect the number of surviving target cells on biocompatible microelectrode surfaces, were calculated using RTCA software every 15 min. The cell index data of each group represent the mean value of 4 wells.

### Quantitative real-time PCR

Real-time quantitative polymerase chain reaction (qPCR) was used to assess the expression levels of the CAR fusion genes according to a previously described protocol. A 153-bp (base pair) fragment containing portions of the CD8 α chain and adjacent CD137 chain was amplified using a forward primer 5′-GGTCCTTCTCCTGTCACTGGTT-3′ and reverse primer 5′-TCTTCTTCTTCTGG AAATCGGCAG-3′ to detect the CAR gene; amplification of GAPDH was used as an internal control and for normalization of DNA quantities. qPCR was performed using Fast All-in-One RT Kit with SYBR Green Master Mix (ES Science) and run on a LightCycler 480 (Roche).

### Western blot analysis

To demonstrate that CD133 CAR-T and PD-1 s cells can secrete PD-1 blocking scFv, CAR-T cells were centrifuged at 2000 × *g* for 20 min at 4 °C. Then, the supernatant of Mock T, CD133 CAR-T, and CD133 CAR-T and PD-1 s cells was concentrated using a Millipore system (10 kd), with centrifugation at 4000* g* for 15–20 min until the remaining liquid was approximately 200 µl. Then, RIPA (Sigma) containing protease inhibitor was added on ice for 15 min, and PD-1 blocking scFv was detected using a rabbit anti-c-Myc-tag antibody (CST, Cat 14,038, 1:1000). Detection of antibody was achieved with Pierce ECL western blot substrate (Tanon). To detect whether the CAR genes integrated into T cells, endogenous CD3 zeta and exo-CD3zeta were detected using anti-CD3 ζ (Santa Cruz Biotechnology, Clone 6B10.2, 1:200).

### ELISA

The supernatants of Mock T cells, CD133 CAR-T cells, and CD133 CAR-T and PD-1 s cells incubated with tumour cells at a ratio of 1:1 for 24 h were collected and stored at − 80 °C. The supernatant was then thawed, and the IFNγ, TNFα and IL-2 concentrations were detected according to the manufacturer’s protocols (Dakewe Biotech).

### Cellular immunofluorescence

The PD-1 + T cells constructed with the lentivirus were washed with cold PBS 2–3 times, and tips were used to place T cells on a slide. The cells were fixed with 4% paraformaldehyde, washed with PBS, permeabilized with 1% Triton X-100 for 10 min at room temperature, blocked with goat serum for 30 min, washed with PBS, incubated with anti-c-Myc-tag-647 directly or incubated with the condensed supernatant from CD133 CAR-T and PD-1 s cells, and then incubated with c-Myc-tag-647. The fluorescence was assessed by using a Nikon Eclipse Ti2.

### In vivo experiments

All experiments were performed in compliance with all relevant ethical regulations and in accordance with an Institutional Animal Care and Use Committee-approved protocol (No. 18080B). NCG mice (GuangDong GemPharmatech Co., Ltd.) aged 4–6 weeks were inoculated subcutaneously with 2.5 × 10^6^ tumour cells (Hep-3B-luc), and after the tumour grew to 50–100 mm^3^, Mock T, CD133 CAR-T and CD133 CAR-T and PD-1 s cells were intravenously injected into NCG mice with Hep-3B. For metastatic mouse models, the NCG mice were intravenously injected with 2.5 million Hep3B-luc cells. The metastasis was confirmed by bioluminescence imaging (BLI) and then treated with a tail veil injection of 5 million Mock T, CD133 CAR-T or CD133 CAR-T and PD-1 s cells. BLI of tumour growth was performed using a PerkinElmer IVIS imaging system with Living Image software. Finally, the tumour weight was evaluated. Mice were euthanized when the tumour volume reached 2000 mm^3^ or when they showed any sign of sickness. For establishment of an in situ xenograft tumour model, 2.5 million Hep3B-luc cells were directly injected into the liver of NCG mice. The tumour was confirmed by BLI, and 5 million Mock T, CD133 CAR-T or CD133 CAR-T and PD-1 s cells were injected into the tail vein. BLI of tumour growth was performed using a PerkinElmer IVIS imaging system with Living Image software.

### Statistical analysis

All statistical analyses were performed using GraphPad Prism Software (GraphPad Software Inc., version 8.02) and SPSS (IBM, version 25.0), except for survival curves, which were generated by the Kaplan–Meier survival method and compared with the log-rank test using the R programming language. Two-tailed unpaired *t* tests were used for comparisons of 2 groups. One-way ANOVA was used for comparisons of 3 or more groups in a single condition. The factors predicting OS and PFS were assessed via univariate and multivariate Cox proportional hazard regression model analyses. Differences were considered statistically significant as follows: *P < 0.05, **P < 0.01 and ***P < 0.001.

## Results

### Clinical characteristics of CD133 and PD-L1 expression in HCC

**Fig. 1:**
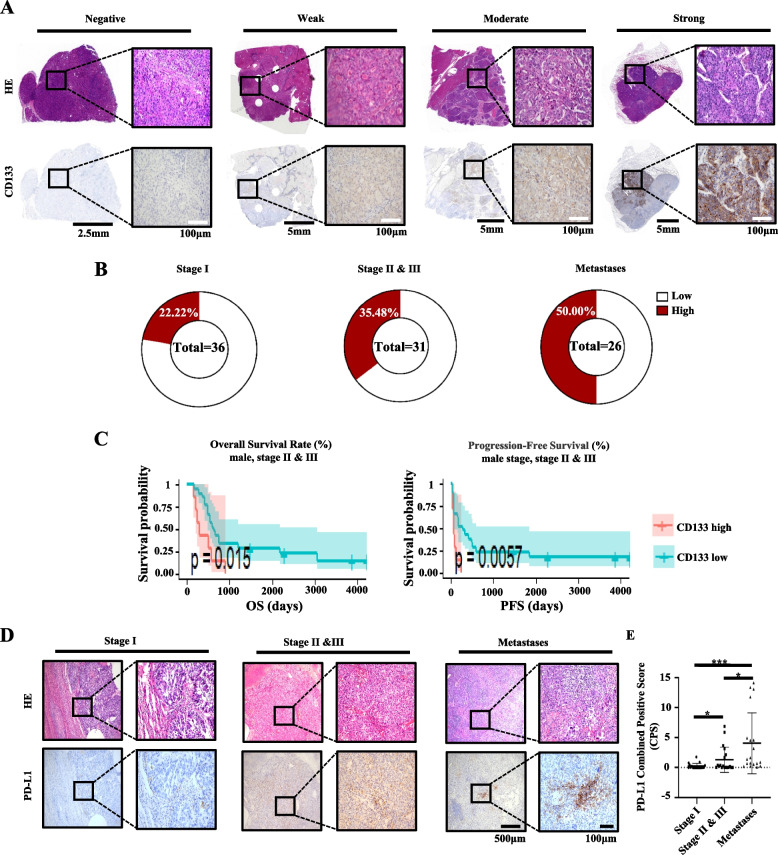
Representative image of CD133 and PD-L1 detection in different stages in HCC patients. **A** HE images and corresponding CD133 expression in primary tumour tissue in liver cancer patients and lung metastases. **B** The percentage of CD133 expression in primary tumours in early (stage I) and advanced stages (stage II and III) and metastases in the lung. **C** Kaplan–Meier curve depicting survival analyses in stage II and III male patients. The *P* value was calculated by the log-rank test. **D** Representative image of PD-L1 expression at different stages and in lung metastases of HCC patients. **E** The PD-L1 expression at different stages and in lung metastases of HCC patients were evaluated using the CPS score method and compared. Mean ± SD, *n* = 19, two-tailed unpaired Student’s *t* test, *P < 0.05, **P < 0.01, ***P < 0.001, ****P < 0.0001, ns, not significant

To evaluate the suitability of CD133 as a target for CAR-T cell therapy in HCC, we first analysed CD133 protein levels in 67 HCC patient samples (stage I, 36 cases; stage II, 7 cases; stage III, 24 cases; stage IV, 26 lung metastasis cases) and matched adjacent tissue by IHC assay. CD133 expression was classified into four levels, negative (0), weak (less than 5), moderate (5–10) and strong (more than 10) (Fig. 1A), and was further divided into CD133-high or CD133-low expression using the median IHC score (2.284). High expression of CD133 could be detected in 19/67 (28.36%) primary HCC tissues, with 8/36 (22.22%) in stage I, 11/31 (35.48%) in stage II and III and 13/26 (50.00%) in the lung metastasis stage (Fig. 1B). Statistical analysis revealed that CD133 was significantly correlated with portal vein invasion and AFP (*P* = 0.002 and *P* = 0.016 respectively; Additional file 2: Table S1). There was no significant association between CD133 expression and age, sex, HBsAg, liver cirrhosis, tumour size, tumour number, tumour encapsulation, histological differentiation, TNM stage or microvascular invasion (Additional file 2: Table S1). We next analysed the impact of CD133 protein expression levels on HCC patient survival. Although there was no significant association between CD133 expression and OS or PFS) in all patients (Additional file 2: Fig. S1A, Table S2), CD133 expression in the late stage (II and III) in male patients was significantly associated with PFS (*P* = 0.0057) and worse OS (*P* = 0.015) in univariate analysis (Fig. 1C and Additional file 2: Tables S3, S4), with a trend toward worse OS in multivariate analysis (*P* = 0.041) (Additional file 2: Table S4). Furthermore, online TCGA data (https://www.proteinatlas.org/ENSG00000007062-PROM1/pathology/liver+cancer) for HCC also showed that CD133 expression in late stage or male patients was correlated with worse OS (Additional file 2: Fig. S1B). These characteristics make the CD133 protein a reasonable target for immunotherapy in male patients with advanced HCC. To evaluate whether male patients with advanced HCC will also benefit from PD-1 checkpoint blockade therapy, we also evaluated PD-L1 expression in male HCC patients using the CPS method. A total of 57 slides (Stage 1, Stages 2 and 3 and lung metastases, 19 slides of each stage) were used to assess PD-L1 expression. Significant higher CPS scores were found for the metastases and Stages II and III samples compared with the Stage I samples (*P* = 0.0024 and *P* = 0.0356, respectively; Fig. 1D, E), which indicated that male patients with advanced HCC may benefit from PD-1/PD-L1 checkpoint blockade therapy.

### The construction of CD133-CAR-T cells secreting PD-1 blocking scFv using the SB transposon system and MC technology

**Fig. 2:**
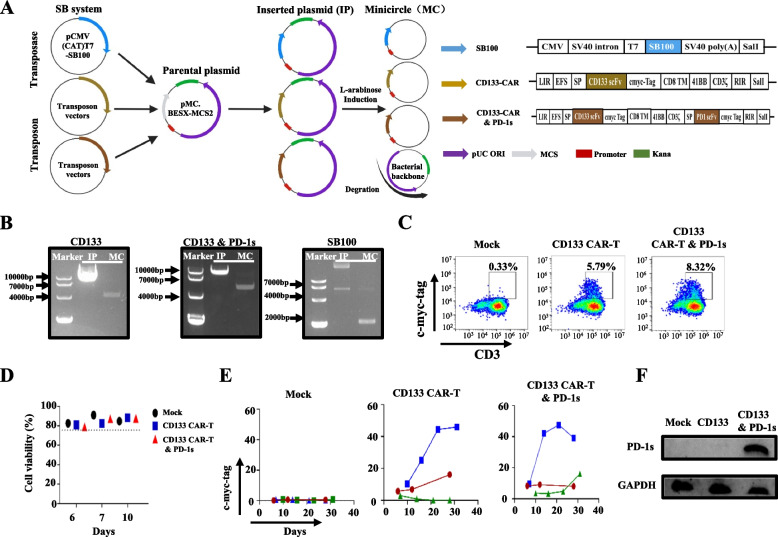
Construction of CD133 CAR-T and PD-1 s cells based on sleeping beauty transposons from minicircles. **A** Schematic diagram of CAR-T cell preparation and sequences of SB100, CD133 CAR-T and CD133 CAR-T and PD-1 s cells. **B** The minicircles from CD133 CAR, CD133 and PD-1 s CAR and SB100 cells were evaluated by agarose gel electrophoresis. **C** Representative flow cytometry analyses of CAR-T cell expression using a c-Myc-tag as shown in the surrogate. **D** The cell viability of CAR-T cells from 3 different donors. **E** CAR expression detected at different time points from 3 different healthy donors. One colour represents one patient sample. **F** Western blot detection of the secreted PD-1 blocking scFv in the supernatant of Mock T cells, CD133 CAR-T cells and CD133 CAR-T and PD-1 s cells

Next, CD133-specific CAR-T cells secreting PD-1 blocking scFv antibody were constructed. We used an SB-based nonviral genetic modification system involving MC transposon donor vectors to simplify the current CAR-T cell production protocol. The MC-encoded transposase was prepared by cutting SB100 from pCMV (CAT)T7-SB100 and inserting it into the MCS clone site of a parental MC cloning vector (pMC.BESPX-MCS2). The MC-encoded transposon was prepared by two transposon vectors expressing optimized CD133-CAR or CD133-CAR and PD-1 blocking scFv with c-Myc transduction markers and inserted into the MCS clone site of pMC.BESPX-MCS2 (Fig. 2A). These plasmids containing MC-encoded transposase and transposons were recombined with L-arabinose for the production of MCs, resulting in a vector size reduction (Fig. 2B). After transfecting transposon and transposase vectors at two ratios (3:1 and 1:1) in T cells from the peripheral blood of healthy donors, we found that a ratio of 1:1 had better transfection efficiency and cell viability. On days 5–10, we observed 5–9% CAR engraftment of T cells with CD133-CAR or CD133-CAR and PD-1 blocking scFv by electrotransfection (Fig. 2C), and cell viability was more than 75%, as assessed by 7-AAD staining (*n* = 3) (Fig. 2D). A ratio of 3:1 resulted in lower cell viability, and the cells could not persist in long-term culture (Additional file 2: Fig. S2A-C). The transfection efficiency was maintained or increased for more than 28 days (Fig. 2E). To demonstrate PD-1 blocking scFv secretion by CD133-specific CAR-T cells, the concentrated supernatant of modified T cells was collected and analysed by western blotting. We found that only the T cells transfected with CD133-CAR and PD-1 blocking scFv could secrete the PD-1 blocking scFv (Fig. 2F). Collectively, these data showed that CD133-specific CAR-T cells secreting PD-1 blocking scFv (CD133 CAR-T and PD-1 s) were constructed successfully based on SB-mediated transposition from MC transposon donor vectors.

### CD133 CAR-T and PD-1 s cells show potent antitumour effects in a CD133 high-expression HCC cell line

**Fig. 3:**
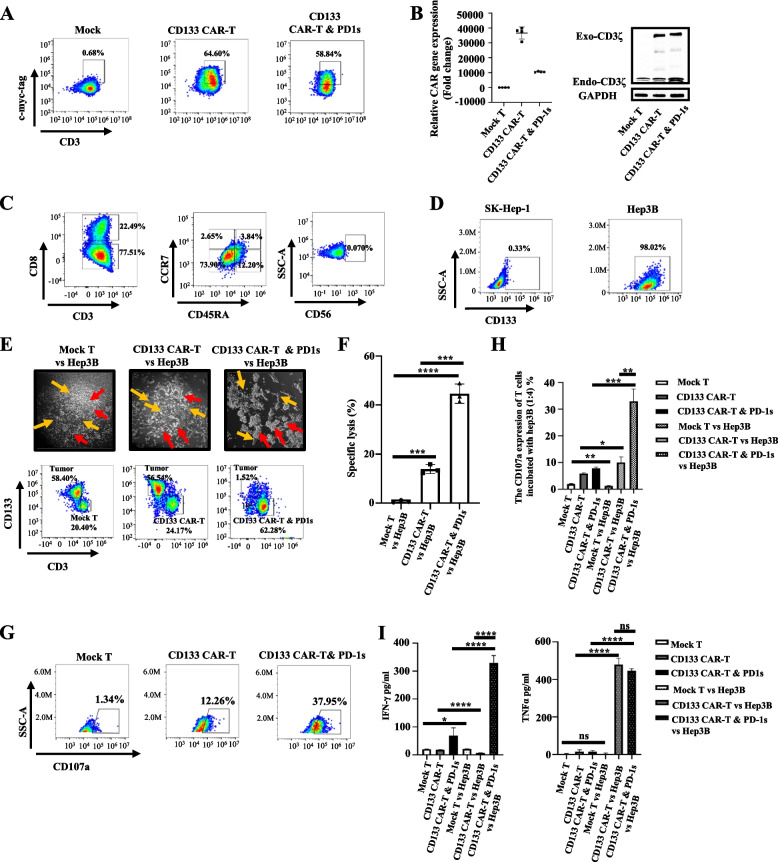
Enhanced specific cytotoxic effects of CD133 CAR-T and PD-1 s cells against CD133 + HCC cell lines. **A** CAR expression in Mock, CD133 CAR-T and CD133 CAR-T and PD-1 s cells was assessed by flow cytometry after enrichment with anti-c-Myc-tag-biotin and anti-biotin microbeads. **B** RT-QPCR and western blot analysis of the CAR gene number and CD3ζ expression in Mock T, CD133 CAR-T, and CD133 CAR-T and PD-1 s cells. **C** The CD8-positive subset of CD133 CAR-T and PD-1 s cells and their CCR7 and CD45R expression by flow cytometric analysis. **D** Flow cytometry analysis of CD133 expression in HCC cell lines (SK-Hep-1 and Hep3B). **E** The upper images show Mock T, CD133 CAR-T and CD133 CAR-T and PD-1 s cells targeting Hep3B cells. The red arrow represents the CAR-T cells, and the yellow arrow represents the tumour. The lower image shows the proportion of Mock T, CD133 CAR-T cells or CD133 CAR-T and PD-1 s cells incubated with CD133 + HCC cell lines (Hep3B) after 24 h. **F** Specific lysis (ratio of 7AAD-positive cells to CD133 + tumour total cells) after 3 days of incubation of CAR-T cells with tumour cells. **G** Representative flow cytometric image of CD107a expression in Mock T cells, CD133 CAR-T cells, and CD133 CAR-T and PD-1 s cells incubated with tumour cells for 4 h. **H** The frequency of CD107a expression on Mock T, CD133 CAR-T, CD133 CAR-T and PD-1 s cells alone or incubated with Hep3B cells by flow cytometric analysis. **I** Cytokine production (IFNγ and TNFα) by Mock T cells, CD133 CAR-T cells, and CD133 CAR-T and PD-1 s cells with or without tumour cell incubation for 4 h was measured by ELISA. Mean ± SD, *n* = 4, two-tailed unpaired Student’s* t* test, *P < 0.05, **P < 0.01, ***P < 0.001, ****P < 0.0001, ns, not significant

We next analysed the antitumour efficacy of CD133 CAR-T and PD-1 s cells. CD133 CAR-T and PD-1 s cells were first incubated with a biotinylated anti-c-Myc-tag antibody and then with anti-biotin microbeads by positive selection. The CAR expression level was more than 50% after 7–11-fold enrichment (Fig. 3A). qPCR and WB further confirmed CAR integration (Fig. 3B). The CD4/CD8 ratio was approximately 3.5:1, and most CAR-T cells were CD45RA-CCR7-T cells (Fig. 3C). The analysis revealed Treg proportions of 38.4 and 1.23% among CD133 CAR-T and CD133 CAR-T and PD-1 s cells, respectively (Additional file 2: Fig. S3), but the CD4 + CD133 CAR-T or CD133 CAR-T and PD-1 s cells still exhibited antitumour ability (Additional file 2: Fig. S4). Two hepatocellular cell lines, SK-Hep1 (1% CD133 expression) and Hep3B (98.02% CD133 expression) (Fig. 3D), were chosen to evaluate cytotoxic activity. The antitumor advantage of CD133 CAR-T and PD-1 s cells was evaluated by continuous cytotoxicity assays with E:T ratios of 1:1, 0.5:1 and 0.25:1 for 72 h using an xCELLigence Real-Time Cell Analyzer-Multiple Plate system (Agilent Technologies). For total T cells, at a low E:T ratio of 0.25:1, the CD133 CAR-T and PD-1 s CAR-T cells exhibited greater cytotoxicity than the CD133 CAR-T cells (*P* = 0.0309) (Additional file 2: Fig. S4A), whereas no differences were observed at E:T ratios of 1:1 and 0.5:1 (*P* = 0.9650 and *P* = 0.1837, respectively) (Additional file 2: Fig. S4A). These results corresponded with those of a previous study showing that PD-1 scFv-secreting CAR-T cells exhibited greater cytotoxicity than conventional CAR-T cells at a low E:T ratio or with increased antigen exposure time [20]. To investigate whether the CD133 CAR-T and PD-1 s cells could prevent CD133 CAR-T cell exhaustion, we compared the cytotoxicity of CD133 CAR-T and PD-1 s cells, CD133 CAR-T cells and Mock T cells at a low E:T ratio of 0.25:1 by flow cytometry. The cytotoxicity of CD133 CAR-T and PD-1 s cells to the CD133-high-expression HCC cell line Hep3B was significantly enhanced compared with that of CD133 specific CAR-T and Mock T cells at an E:T of ratio 0.25:1 overnight (*P* = 0.002 and *P* < 0.0001; Fig. 3E, F). In addition, the percentage of CD107a + T cells (*P* = 0.0014; Fig. 3G, H) and the production of IFNγ (*P* < 0.0001; Fig. 3I) were increased in CD133 CAR-T and PD-1 s cells compared with CD133-specific CAR-T cells. Overall, CD133 CAR-T and PD-1 s cells have better cytotoxicity than CD133-specific CAR-T cells in vitro.

We further explored the effect of PD-1 scFv secretion on CD8 + and CD4 + T cells purified from the total CAR-T cell population. For the CD8 + T cells, the CD133 CAR-T and PD-1 s CAR-T cells exhibited greater cytotoxicity than the CD133 CAR-T cells at all E:T ratios (*P* = 0.0490, *P* = 0.0003 and *P* = 0.0007, respectively) (Additional file 2: Fig. S4B). For the CD4 + T cells, the CD133 CAR-T and PD-1 s CAR-T cells showed greater cytotoxicity than the CD133 CAR-T cells at an E:T ratio of 0.25:1 (*P* = 0.0094) (Additional file 2: Fig. S4C), whereas no differences were observed at E:T ratios of 1:1 and 0.5:1 (*P* = 0.9200 and *P* = 0.8012, respectively) (Additional file 2: Fig. S4C).

The expression of TNFα, IFNγ and CD107a at an E:T ratio of 0.25:1 was monitored in vitro. The expression of IFNγ and CD107a in both the CD8 + and CD4 + T cells of CD133 CAR-T and PD-1 s cells was higher than that of CD133 CAR-T cells (*P* < 0.0001, *P* < 0.0001 for CD8 + T cells; *P* = 0.009, *P* < 0.0001 for CD4 + T cells) (Additional file 2: Fig. S5A-C). The expression of TNFα in the CD8 + T cells of CD133 CAR-T and PD-1 s was higher than that of CD133 CAR-T cells (*P* = 0.0003) (Additional file 2: Fig. S5C). However, no differences in the expression of TNFα was found between the CD4 + T cells of CD133 CAR-T and PD-1 s cells and the CD4 + T cells of CD133 CAR-T cells when incubated with Hpe3B cells (*P* = 0.1087) (Additional file 2: Fig. S5C).

### CD133 CAR-T and PD-1 s cells can bind bystander PD-1 T cells

**Fig. 4:**
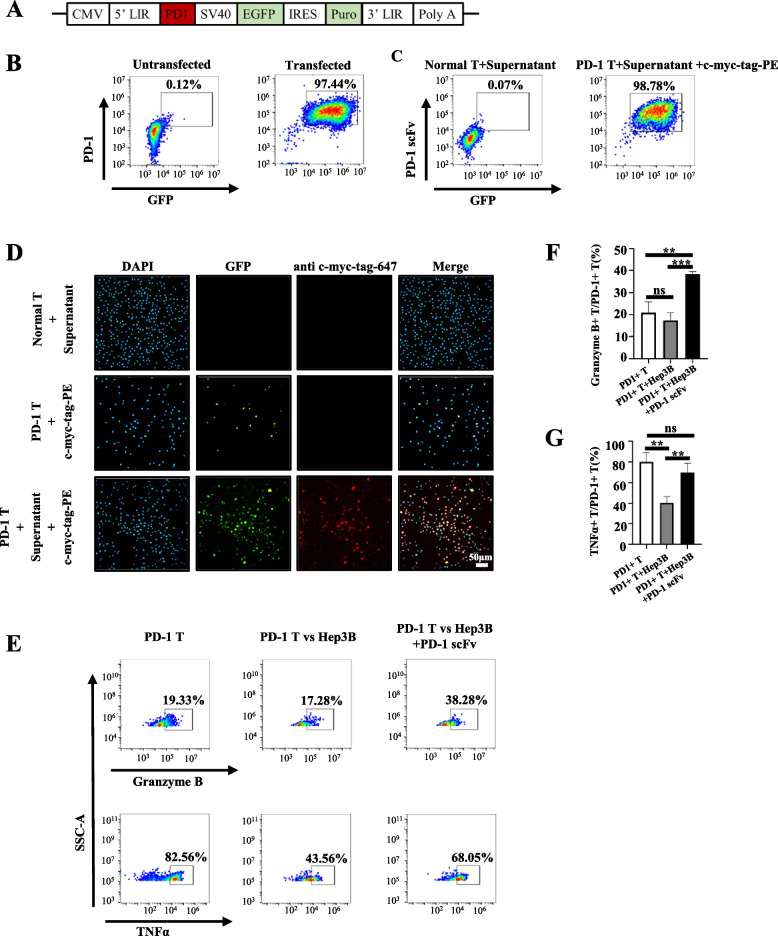
The PD-1 blocking scFv secreted by CD133 CAR-T and PD-1 s can bind bystander PD-1 + T cells and restore T cell function. **A** Schematic diagram of the PD-1 overexpression lentivirus with a GFP flag and a puromycin selection marker. **B** Flow cytometry analysis of PD-1 and GFP expression in T cells or T cells transfected with a PD-1 overexpression lentivirus and then selected with puromycin. **C** Flow cytometry analyses of the secreted PD-1 blocking scFv binding to normal T cells or PD-1 T cells. **D** Cellular immunofluorescence assay of PD-1 (GFP) and PD-1 blocking scFv (anti c-Myc-tag-647, Red) expression on normal T cells, PD-1 T cells, and PD-1 T cells incubated with the condensed supernatant from CD133 CAR-T and PD-1 s cells. **E** Representative flow cytometric images of granzyme B and TNFα expression in PD-1 T cells, PD-1 T cells incubated with Hep3B, and PD-1 T cells incubated with Hep3B and PD-1 blocking scFv. **F**, **G** Quantification of the frequency of granzyme B/PD-1 T cells and TNFα/PD-1 T cells by flow cytometric analysis of PD-1 + T cells, PD-1 + T cells incubated with Hep3B cells and PD-1 + T cells incubated with Hep3B cells and PD-1-blocking scFv secreted by CD133 CAR-T and PD-1 s cells. Mean ± SD, *n* = 3, two-tailed unpaired Student’s* t* test, *P < 0.05, **P < 0.01, ***P < 0.001, ns, not significant

To demonstrate that bystander T cells can benefit from the PD-1 blocking scFv secreted from nearby CD133 CAR-T and PD-1 s cells, we first introduced PD-1 into healthy donor T cells using a lentiviral vector followed by EGFP and puromycin selection (Fig. 4A) and obtained more than 97% PD-1-expressing T cells (Fig. 4B). The culture medium from CD133 CAR-T and PD-1 s cells was concentrated and cocultured with normal T cells and PD-1-expressing T cells for 30 min. Flow cytometry analysis demonstrated that secreting PD-1 blocking scFv can bind to PD-1 on PD-1-expressing T cells but not normal T cells (Fig. 4C). These results confirmed that the PD-1 blocking scFv secreted by CAR-T cells can bind to bystander PD-1 + T cells. Immunofluorescence also confirmed these results (Fig. 4D). To further demonstrate that the secreted PD-1 blocking scFv could restore the function of PD-1 + T cells, the PD-1 + T and Hep3B cells were incubated with or without PD-1 blocking scFv. The granzyme B and TNFα levels were significantly higher in the presence of PD-1 blocking scFv compared with those of PD-1 + T cells and Hep3B in the absence of PD-1 blocking scFv (*P* = 0.0006 and *P* = 0.0094; Fig. 4E–G).

### Antitumour ability of CD133 CAR-T and PD-1 s cells in vivo

**Fig. 5:**
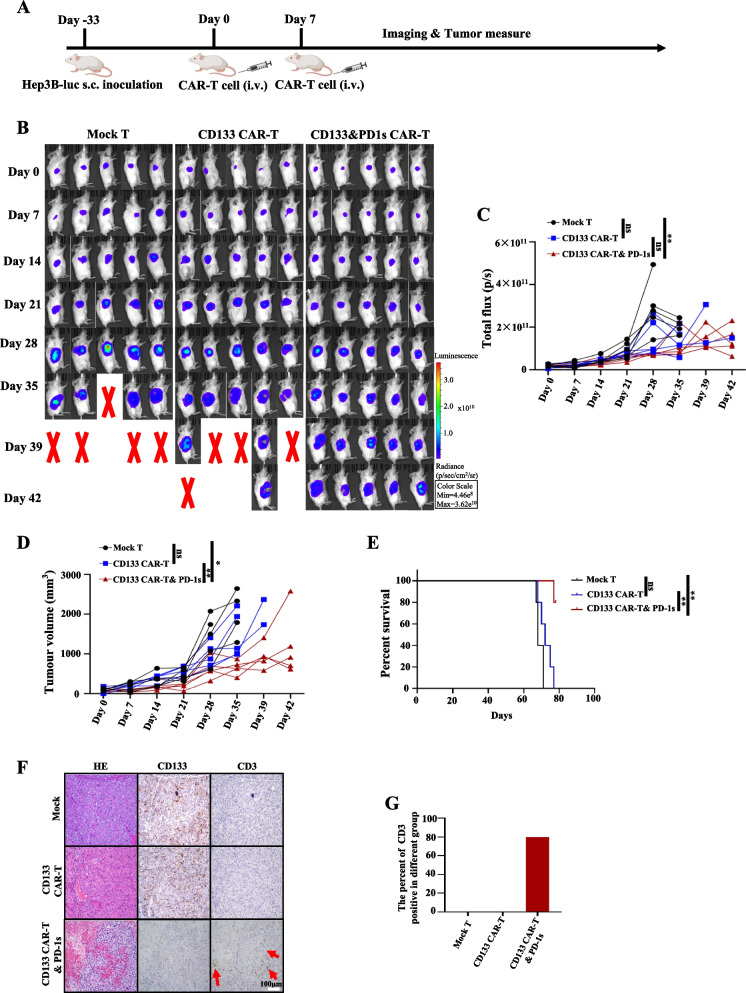
CD133 CAR-T and PD-1 s cells have better antitumour effects than CD133 CAR-T cells in vivo after intravenous injection. **A** Schematic diagram of CAR-T cell treatment and tumour detection. NCG mice with Hep3B xenograft tumours were intravenous injected with two doses of 5 million Mock T cells, CD133 CAR-T cells or CD133 CAR-T and PD-1 s cells once Hep3B xenograft tumours reached 50–150 mm^3^, as revealed by bioluminescence imaging. **B** The tumours of Mock T cell-, CD133 CAR-T cell- and CD133 CAR-T and PD-1 s cell-treated groups were monitored weekly by bioluminescence imaging. **C** Growth curves of Mock T cell-, CD133 CAR-T cell- and CD133 CAR-T and PD-1 s cell-treated groups in Fig. 5B. **D** The tumour volumes of the Mock T cell-, CD133 CAR-T cell- and CD133 CAR-T and PD-1 s cell-treated groups were measured by bioluminescence imaging. Mean ± SD, *n* = 5, two-tailed unpaired Student’s *t* test, *P < 0.05, **P < 0.01, ***P < 0.001, ns, not significant. **E** Kaplan–Meier survival curves of Mock T, CD133 CAR-T and CD133 CAR-T and PD-1 s cell-treated groups. **P < 0.01 (log-rank test); ns, not significant. **F** Representative images of HE, CD133 and CD3 staining of the Mock T, CD133 CAR-T and CD133 CAR-T and PD-1 s cell-treated groups. **G** Percentage of CD3 expression in tumours in all the mice in Fig. 5F belonging to the Mock T, CD133 CAR-T and CD133 CAR-T and PD-1 s cell-treated groups

**Fig. 6:**
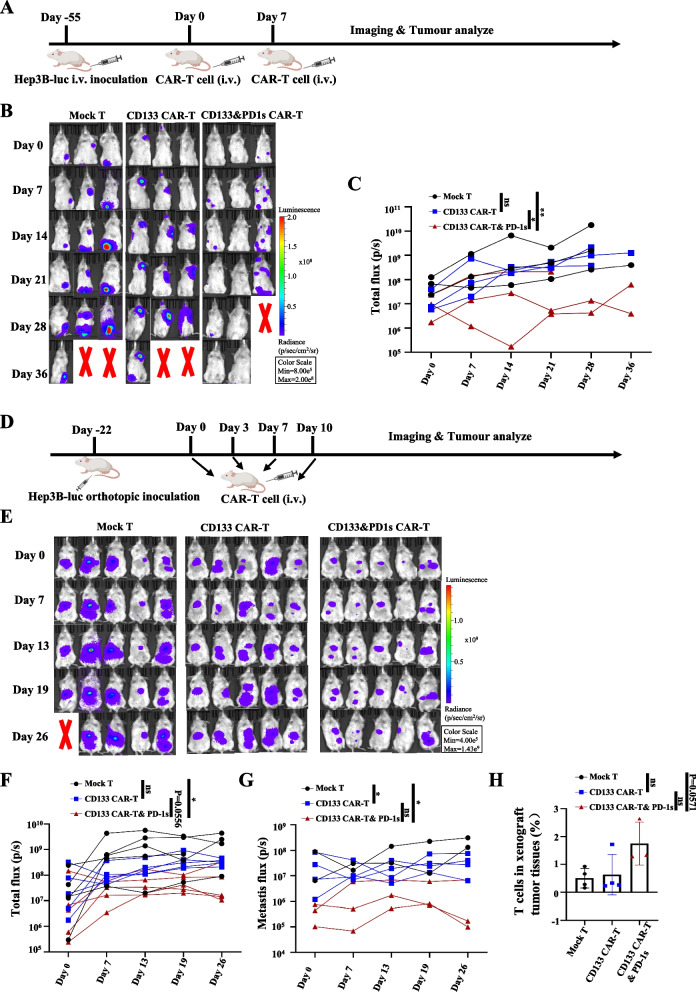
CD133 CAR-T and PD-1 s cells eliminate tumour cells in a Hep3B metastatic and in situ xenograft tumour mouse models. **A** Schematic diagram of CAR-T cell treatment. In brief, NCG mice were intravenously injected with 2.5 million Hep3B-luc cells. Metastasis was confirmed by bioluminescence imaging, and the groups were then treated with a tail veil injection of 5 million Mock T, CD133 CAR-T, or CD133 CAR-T and PD-1 s cells 55 days after inoculation. **B** Bioluminescence imaging of Mock T cell-, CD133 CAR-T cell- or CD133 CAR-T and PD-1 s cell-treated groups in a metastatic model of HCC. **C** The growth curves of the Mock T cell-, CD133 CAR-T cell- and CD133 CAR-T and PD-1 s cell-treated groups are shown in Fig. 6B. Mean ± SD, *n* = 3, two-tailed unpaired Student’s *t* test, **P < 0.01, ns, not significant. **D** Schematic diagram of CAR-T cell treatment. In brief, 2.5 million Hep3B-luc cells were injected into the livers of NCG mice. The tumour was confirmed by bioluminescence imaging and then treated with a tail veil injection of 5 million Mock T, CD133 CAR-T or CD133 CAR-T and PD-1 s at 22 days after inoculation. **E** The bioluminescence imaging of Mock T cell-, CD133 CAR-T cell- or CD133 CAR-T and PD-1 s cell-treated groups in an situ xenograft tumour model of HCC. Mean ± SD, *n* = 5, two-tailed unpaired Student’s *t* test, *P < 0.05, ns, not significant. **F**, **G** The total flux and the metastasis flux of the Mock T cell-, CD133 CAR-T cell- and CD133 CAR-T and PD-1 s cell-treated groups in Fig. 6E. Mean ± SD, *n* ≥ 2, two-tailed unpaired Student’s *t* test, **P < 0.01, ns, not significant. **H** The bar graph shows the frequency of CAR-T cells 16 days after the last instance of Mock T, CD133 CAR-T and CD133 CAR-T and PD-1 s cell therapy identified by flow cytometry using anti-human CD3

To test the in vivo efficacy of CD133 CAR-T and PD-1 s cells, we injected Hep3B cancer cells subcutaneously into NCG mice to establish xenograft tumours. CD133 CAR-T and PD-1 s cells, CD133-specific CAR-T and Mock T cells were applied intravenously on day 0 and day 7 at a dose of 5 million T cells (Fig. 5A). BLI of the CD133 CAR-T and PD-1 s cell-treated group was much lower than that of the Mock T cell-treated group on day 28 (*P* = 0.0159; Fig. 5B, C). The tumour volume measured by BLI of the CD133 CAR-T and PD-1 s cell group was smaller than that of the CD133-specific CAR-T and Mock T-cell-treated groups on day 21 (*P* = 0.0079 and *P* = 0.0159, respectively; Fig. 5D). The survival analysis also revealed that the CD133 and PD1s CAR-T cells significantly prolonged mice survival compared with CD133 CAR-T cells and Mock T cells (*P* = 0.0044 and *P* = 0.0026, respectively; Fig. 5E). To evaluate the number of human T cells inside mouse xenograft tumours, we used IHC staining of xenograft tumours with a human CD3 antibody. CD3 staining was higher in CD133 CAR-T and PD-1 s cell-treated mice than in CD133-specific CAR-T-treated and Mock T-cell-treated mice (Fig. 5F). To evaluate the number of human T cells inside mouse xenograft tumours, we performed IHC staining of xenograft tumours with human CD3 antibody. The CD3-stained area in CD133 CAR-T and PD-1 s cell-treated mice (4/5) was higher than that in CD133-specific CAR-T-treated (0/5) and Mock T-cell-treated mice (0/5) (Fig. 5F, G).To evaluate the antitumour efficiency of CD133 and PD1s CAR-T cells in a metastasis mouse model, a metastasis xenograft mouse model was established by injecting 2.5 million transfected Hep3B-luc cells into NCG mice until BLI showed a stable tumour followed by administering T cell therapy 55 days later (Fig. 6A). The BLI-based growth curve showed that CD133 CAR-T and PD-1 s cell-treated group exhibited better antitumour effects than the CD133 CAR-T cell and Mock T cell-treated group (*P* = 0.0260 and *P* = 0.0087, respectively; Fig. 6B, C).

To further examine the antitumor ability of CD133 CAR-T and PD-1 s cells in an orthotopic HCC mouse model, we injected Hep3B cells into the liver via direct intrahepatic injection to establish an in situ xenograft tumour that mimics the natural liver microenvironment and metastatic HCC. Twenty-two days after tumour inoculation, four doses of CD133 CAR-T and PD-1 s cells were infused intravenously injection (i.v.) to a total of 5 × 10^6 CAR-T cells. The tumour BLI showed that the antitumor ability of CD133 CAR-T and PD-1 s cells was stronger than that of Mock T or CD133 CAR-T cells (*P* = 0.0317 and *P* = 0.0556, respectively, Fig. 6D–G). A higher percentage of human T cells was detected in the tumour microenvironment of the CD133 CAR-T and PD-1 s group than in that of the Mock T group (*P* = 0.0571, Fig. 6H).

## Discussion

Suitable tumour-specific antigens are essential for the design and execution of CAR-T immunotherapy. A series of tumour-specific antigens, such as Glypican 3 (GPC3), epidermal growth factor receptor variant III (EGFRvIII), c-Met, PD-L1, CD133, NK group 2 member D (NKG2D) ligands (NKG2DL) and some viral-derived cancer antigens, have been explored and used to construct CAR-T cells against HCC [21–23]. Moreover, some tumour antigen-specific CAR-T cells have finally entered clinical trials; five clinical trials investigated glypican-3, and other less conventional targets, including EpCAM, claudin 18.2, DR5, c-met, EGFR*vIII* and CD133, were also explored (*NCT03013712, NCT03302403, NCT03638206, NCT03941626, NCT02541370*). Among these tumour antigens, cytoplasmic CD133 reportedly corresponds with poor prognostics in human HCC patients [24]. CD133 is known to be expressed on somatic stem cell populations. Beyond the haematopoietic [25] and neural [26] systems, CD133 is physiologically expressed in ductal epithelia of glandular organs, which are proposed to host cells with dedifferentiation capacities [27]. Thus, the targeting of CD133 + on normal body cells might bear the risk of potential side effects. However, the expression of CD133, which is typically present on CD34-enriched normal haematopoietic system progenitor cells, is very low (< 5000 sites per cell) [28], and a humanized mouse model was used to test the toxicity of anti-CD133 immunotherapies against a human haematopoietic stem cell niche [29], which demonstrated that the proportion of CD133-specific CAR-T cells among the total population of CD34 + haematopoietic stem and progenitor cells (HSPCs) is minimal. In addition, CD133 shows limited staining in normal epithelial cells in ductal epithelia [30]. More importantly, CD133-directed CAR-T has been assessed in a phase 1 and phase 2 clinical study (NCT02541370) (23 patients, including 14 with HCC), which showed that primary toxicity due to a decrease in the haemoglobin/platelet ratio (Grade 3) showed self-recovery within 1 week. The most common high-grade adverse event was hyperbilirubinemia. These results provided preliminary evidence showing that CART-133 cells exhibit manageable safety in advanced HCC. In this study, we demonstrated that total CD133 expression was associated with poor prognosis in male patients with advanced HCC, which was consistent with online TCGA data. Our results further confirmed that CD133 was an attractive and reasonable target for CAR-T cells in HCC.

To the best of our knowledge, CD133 CAR-T cells are usually constructed using lentiviruses or retroviruses, which are lengthy and expensive [16, 31–33]. In addition, viral vectors may also cause potential side effects, including potential carcinogenic, immunogenicity, inflammatory responses and adverse genomic changes [34]. The SB transposon system, comprising two essential components, the transposase enzyme and transposon DNA, is an efficient nonviral gene transfer tool in mammalian cells [35, 36]. The comparable transfection efficacy for cells generated by virus-based gene transfer with the added benefits of lower immunogenicity, enhanced safety profiles, reduced manufacturing complexity and vector procurement cost which reported in previous study make the SB transposon system an attractive system to construct CAR-T cells [37–39]. CAR-T cells targeting CD19 based on SB transposons have entered clinical studies and showed no severe toxicity [40–42], but at present, this technology does not take inhibitory receptors (such as PD-1) of T cells into account. In this study, we used the Sleeping Beauty system to construct PD-1-blocking scFv CD133 specific CAR-T cells against HCC for the first time. In combination, the MC platform, which is devoid of bacterial sources of replication and antibiotic resistance genes, can greatly enhance SB transposition and transgene integration, resulting in higher numbers of stably modified target cells [39].

The tumour microenvironment is another challenge affecting CAR-T cell therapy efficacy. The tumour microenvironment can induce inhibitory receptors on T cells, especially PD-1 [43]. The combination of CAR-T cells and PD-1 seems more promising. For example, ICAM1-targeted CAR T cells in combination with PD-1 blockade demonstrated an improved ability to eradicate ICAM1-expressing target tumour cells compared to CAR T treatment alone [44]. A phase I trial of regional mesothelin-targeted CAR-T cell therapy in combination with the anti-PD-1 agent pembrolizumab in patients with malignant pleural disease was reported, and the median overall survival of combination therapy was 23.9 months, while only CAR-T therapy was 17.7 months [45]. Nevertheless, systemic administration of PD-1 blockade may cause side effects called immune-related adverse events (IRAEs) [46]. Thus, several strategies have been explored to block the inhibitory receptor PD-1 on T cells, and these strategies include coexpression of a chimeric switch receptor, with the extracellular domain of PD-1 linked to activation signalling domains, comodelling of CAR-T cells to express a dominant negative PD-1 receptor [47], cotransduction of CAR-T cells with vectors expressing PD-1-targeting shRNAs [48] and use of the CRISPR/Cas9 gene-editing system to disrupt PD-1 on glypican-3 (GPC3)-targeted second-generation CAR-T cells [49]. However, the protective effect is limited to the CAR-T cells themselves. Endogenous TILs within the tumour microenvironment are still subject to PD-1-mediated suppression. Therefore, given that the PD-1-blocking scFv secreted by CAR-T cells was able to bind to bystander cells, we hypothesize that local secretion of CD133-specific CAR-T cells secreting PD-1 blocking scFv cells in this study may protect endogenous antitumour immune cells and reduce the outgrowth of CD133-negative tumours. Although using SB plasmids from minicircles for the transfection of T cells to construct CAR-T cells was attractive, only 8% of CD133 CAR-T and PD-1 s cells were acquired without enrichment through c-Myc-tags in our study, and this efficiency was similar to that in other studies [39, 50]. The enrichment procedure increases the length of in vitro culture time, which adds the risk of contamination in in vitro culture, and takes more time to acquire enough cells for clinical application. In addition, the length of in vitro culture time also led to reduced or absence of stem cell memory T cells (Tscm) or central memory T cells (TCM) which was reported to achieve durable clinical response for CAR-T cell therapy [51]. Therefore, we need to further improve the transfection of T cells to construct CAR-T cells based on the SB system. One strategy is to explore in vivo amplification, such as using nanotechnology to realize CAR-T cell construction in the blood or using vaccines to expand CAR-T cells in vivo [52–54]. In the future, improving the amplification and persistence of CAR-T cells in vivo may be a promising strategy for CAR-T cells based on SB transposons.

The work presented here provides support for the strategy of using an SB-based nonviral genetic modification system involving MC transposon donor vectors to simplify the current CAR-T cell production protocol, and these CAR-T cells can be utilized to deliver immune modulatory PD-1 scFv within the tumour microenvironment of HCC. This approach could potentially improve the clinical outcome in response to CAR-T cell therapy and improve the safety of checkpoint blockade therapy.

Most of the current studies tested human CAR-T cells in immunodeficient animals, typically NOD Scid Gamma (NSG) mice transplanted with human tumours. The use of NSG mice as human xenograft recipients allows the growth of almost all types of primary human tumours in vivo, including most solid tumours and haematological malignancies that maintain characteristics of the primary tumour in the patient. In addition, the treatment of NSG animals bearing human tumours with CAR-T cells can be used to analyse the antitumour efficacy of CAR-T cells and the ability of these cells to engraft and persist in the blood and tumours of treated animals [55]. However, the use of these animals still has several limitations: (1) studying the interaction between CAR-T cells and the human innate system is difficult, (2) NSG animals do not recreate the immunosuppressive tumour microenvironment in tumour xenografts, and (3) NSG mice fail to reproduce the severe toxicity observed in some clinical trials testing CAR-T cells. Nonetheless, despite these limitations, the NSG model has proven to be useful for further assessment of the direct killing effect of CAR-T cells on tumours. Another limitation of this study is the unequal gender sizes in the cohort. This disparity is a consequence of the higher prevalence of liver cancer in men. Consequently, the number of female patients available for analysis was limited to only 6 out of 67 patients. Given the limited number of female patients, we were unable to identify any immediate differences between male and female patients in our analysis. However, we recognize that this gender imbalance could potentially influence the study results and conclusions. To address this limitation and gain a deeper understanding of CD133 expression and its prognostic implications in liver cancer, further research with a larger and balanced gender cohort is essential.

## Conclusions

In this study, we found that CD133 expression in male patients at the late stage (II and III) was significantly associated with worse PFS and OS. In addition, advanced HCC patients exhibit higher PD-L1 expression compared with patients with early stage HCC, which make the CD133 protein a reasonable target in CAR-T cell therapy for male patients with advanced HCC, and these patients may also benefit from PD-1/PD-L1 checkpoint blockade therapy. Therefore, we used a nonviral method to successfully construct CD133-specific CAR-T cells that could secrete PD-1 scFv checkpoint blockade inhibitors based on a SB system from minicircle vectors, which exhibits less immunogenicity, has a lower cost and is markedly safer compared with viral vectors. We further confirmed the effect of treatment with CD133 CAR-T and PD-1 s cells in vitro and in vivo. Collectively, our preclinical results indicated that CD133 CAR-T and PD-1 s cells may be a therapeutically tractable strategy for male patients with advanced HCC and high CD133 expression.

### Supplementary Information

**Additional file 1.** The sequence of pSB-EF1alpha-antiCD133 CD8 4-1BB CD3z-P2A-PD-1 blocking scFv-WPRE-BGH polyA.

**Additional file 2: Table S1.** Correlation between CD133 expression and clinical characteristics in HCC. **Table S2.** Correlations between clinical characteristics and patient prognosis. **Table S3.** Univariate and multivariate analyses of clinical variables associated with PFS in male patients. **Table S4.** Univariate and multivariate analyses of clinical variables associated with OS in male patients. **Figure S1.** Survival analyses using different evaluation methods. **Figure S2.** Cell viability and CAR expression after transfection with transposon and transposase vectors at a ratio of 3:1 in T cells from PBMCs. **Figure S3.** Treg (CD4+CD25+Foxp3) phenotype of CD4-positive CAR T cells. **Figure S4.** Specific cytotoxic effects of CD133 CAR-T and PD-1s cells against CD133+ HCC cell lines. **Figure S5.** Enhanced specific cytotoxic effects of CD133 CAR-T and PD-1s cells among CD8- or CD4-positve CAR-T cells against CD133+ HCC cell lines.

**Additional file 3.** Images of the original, uncropped gels/blots in Fig. 2 B, F and Fig. 3B.

## Data Availability

The datasets used and analysed during the current study are available from the corresponding author on reasonable request.

## Abbreviations

BGH: Bovine growth hormone
BLI: Bioluminescence imaging
CAR-T: Chimeric antigen receptor-specific T
CD133 CAR-T and PD-1s: CD133-specific CAR-T cells secreting PD-1 blocking scFv
CPS: Combined positive score
CSCs: Cancer stem cells
E:T: Effector to target
EGFRvIII: Epidermal growth factor receptor variant III
GPC3: Glypican 3
HCC: Hepatocellular carcinoma
HSPCs: Haematopoietic stem and progenitor cells
i.v.: Intravenously injection
IRAEs: Immune-related adverse events
ITRs: Inverted terminal repeats
MC: Minicircle
NKG2DL: NK group 2 606 member D (NKG2D) ligands
NSG: NOD Scid Gamma
OS: Overall survival
PD-1: Programmed cell death protein 1
PFS: Progression-free survival
SB: Sleeping Beauty
ScFv: Single-chain variable fragment
TCM: Central memory T cells
Tscm: Stem cell memory T cells
WPRE: Woodchuck hepatitis virus posttranscriptional regulatory element
