# Supplementary material for: Targeted delivery of a PD-1-blocking scFv by CD133-specific CAR-T cells using nonviral Sleeping Beauty transposition shows enhanced antitumour efficacy for advanced hepatocellular carcinoma
Source: BMC Med. 2023 Aug 28;21:327. doi: 10.1186/s12916-023-03016-0 (PMC10464109; doi:10.1186/s12916-023-03016-0)
Supplement: Supplementary file 1 — Additional file 1. The sequence of pSB-EF1alpha-antiCD133 CD8 4-1BB CD3z-P2A-PD-1 blocking scFv-WPRE-BGH polyA. [file 12916_2023_3016_MOESM1_ESM.docx]

**Targeted delivery of a PD-1-blocking scFv by CD133-specific CAR-T cells using nonviral Sleeping Beauty transposition shows enhanced antitumor efficacy for advanced hepatocellular carcinoma**

Chaopin Yang, Jinqi You, Qiuzhong Pan, Yan Tang, Liming Cai, Yue Huang, Jiamei Gu, Yizhi Wang, Xinyi Yang, Yufei Du, Dijun Ouyang, Hao Chen, Haoran Zhong, Yongqiang Li, Jieying Yang, Yulong Han, Fengze Sun, Yuanyuan Chen, Qijing Wang, Desheng Weng, Zhongqiu Liu, Tong Xiang and Jianchuan Xia

The sequence of pSB-EF1alpha-antiCD133 CD8 4-1BB CD3z-P2A-PD-1 blocking scFv-WPRE-BGH polyA was attached bellowed.

actagtgaattccggatccctatacagttgaagtcggaagtttacatacacttaagttggagtcattaaaactcgtttttcaactactccacaaatttcttgttaacaaacaatagttttggcaagtcagttaggacatctactttgtgcatgacacaagtcatttttccaacaattgtttacagacagattatttcacttataattcactgtatcacaattccagtgggtcagaagtttacatacactaagttgactgtgatcaactttgtatagaaaagttgggctccggtgcccgtcagtgggcagagcgcacatcgcccacagtccccgagaagttggggggaggggtcggcaattgaaccggtgcctagagaaggtggcgcggggtaaactgggaaagtgatgtcgtgtactggctccgcctttttcccgagggtgggggagaaccgtatataagtgcagtagtcgccgtgaacgttctttttcgcaacgggtttgccgccagaacacaggtaagtgccgtgtgtggttcccgcgggcctggcctctttacgggttatggcccttgcgtgccttgaattacttccacctggctgcagtacgtgattcttgatcccgagcttcgggttggaagtgggtgggagagttcgaggccttgcgcttaaggagccccttcgcctcgtgcttgagttgaggcctggcctgggcgctggggccgccgcgtgcgaatctggtggcaccttcgcgcctgtctcgctgctttcgataagtctctagccatttaaaatttttgatgacctgctgcgacgctttttttctggcaagatagtcttgtaaatgcgggccaagatctgcacactggtatttcggtttttggggccgcgggcggcgacggggcccgtgcgtcccagcgcacatgttcggcgaggcggggcctgcgagcgcggccaccgagaatcggacgggggtagtctcaagctggccggcctgctctggtgcctggtctcgcgccgccgtgtatcgccccgccctgggcggcaaggctggcccggtcggcaccagttgcgtgagcggaaagatggccgcttcccggccctgctgcagggagctcaaaatggaggacgcggcgctcgggagagcgggcgggtgagtcacccacacaaaggaaaagggcctttccgtcctcagccgtcgcttcatgtgactccacggagtaccgggcgccgtccaggcacctcgattagttctcgagcttttggagtacgtcgtctttaggttggggggaggggttttatgcgatggagtttccccacactgagtgggtggagactgaagttaggccagcttggcacttgatgtaattctccttggaatttgccctttttgagtttggatcttggttcattctcaagcctcagacagtggttcaaagtttttttcttccatttcaggtgtcgtgacaagtttgtacaaaaaagcaggctacgcgtgccaccatgcttctcctggtgacaagccttctgctctgtgagttaccacacccagcattcctcctgatcccactcgacattgttctctcccagtctccagcaatcatgtctgcatctccaggggagaaggtcaccatatcctgcagtgccagctcaagtgtaagttatatgtactggtaccagcagaagccaggatcctcccccaaaccctggatttatcgcacatccaacctggcttctggagtccctgctcgcttcagtggcagtgggtctgggacctcttactctctcacaatcagcagcatggaggctgaagatgctgccacttattactgccagcagtatcatagttacccacccacgttcggtgctgggaccaagctggagctgaaatcctctggtggcggtggctcgggcggtggtgggggtggttcctctagatcttccctcgaggtgaagctggtggagtctggacctgagctgaagaagcctggagagacagtcaagatctcctgcaaggcttctggttataccttcacagactattcaatgcactgggtgaatcaggctccaggaaagggtttaaagtggatgggctggataaacactgagactggtgagccatcatatgcagatgacttcaagggacggtttgccttctctttggaaacctctgccagcactgcctatttgcagatcaacaacctcaaaaatgaggacacggctacatatttctgtgctaccgattacggggactactttgactactggggccaaggcaccactctcacagtctcctcagccaaaacgacacccccatctgtcactagtgagcagaaactcatctctgaagaggatctggctagcctgagcaactccatcatgtacttcagccacttcgtgccggtcttcctgccagcgaagcccaccacgacgccagcgccgcgaccaccaacaccggcgcccaccatcgcgtcgcagcccctgtccctgcgcccagaggcgtgccggccagcggcggggggcgcagtgcacacgagggggctggacttcgcctgtgatatctacatctgggcgcccttggccgggacttgtggggtccttctcctgtcactggttatcaccctttactgcaaacggggcagaaagaaactcctgtatatattcaaacaaccatttatgagaccagtacaaactactcaagaggaagatggctgtagctgccgatttccagaagaagaagaaggaggatgtgaactgagagtgaagttcagcaggagcgcagacgcccccgcgtaccagcagggccagaaccagctctataacgagctcaatctaggacgaagagaggagtacgatgttttggacaagagacgtggccgggaccctgagatggggggaaagccgagaaggaagaaccctcaggaaggcctgtacaatgaactgcagaaagataagatggcggaggcctacagtgagattgggatgaaaggcgagcgccggaggggcaaggggcacgatggcctttaccagggtctcagtacagccaccaaggacacctacgacgcccttcacatgcaggccctgccccctcgcgggcccttcgaaggaagcggagccacgaacttctctctgttaaagcaagcaggagatgttgaagaaaaccccgggcctatgcttctcctggtgacaagccttctgctctgtgagttaccacacccagcattcctcctgatcccacagggccagctggtgcagagcggcgccgaggtgaagaagcccggcgccagcgtgaaggtgagctgcaaggccagcggctacaccttcaccgactacgagatgcactgggtgagacaggcccccggccagggcctggagtggatgggcgtgatcgagagcgagaccggcggcaccgcctacaaccagaagttcaagggcagagccaagatcaccgccgacaagagcaccagcaccgcctacatggagctgagcagcctgagaagcgaggacaccgccgtgtactactgcaccagagagggcatcaccaccgtggccaccacctactactggtacttcgacgtgtggggccagggcaccaccgtgaccgtgagcagcggaggtggtggatccgatgtggtgatgacccagagcccgctgagcctgccggtgaccctgggccagccggcgagcattagctgccgcagcagccagagcattgtgcatagcaacggcaacacctatctggaatggtatctgcagaaaccgggccagagcccgcagctgctgatttataaagtgagcaaccgctttagcggcgtgccggatcgctttagcggcagcggcagcggcaccgattttaccctgaaaattagccgcgtggaagcggaagatgtgggcgtgtattattgctttcagggcagccatgtgccgctgacctttggccagggcaccaaactggaaattaaagagcagaaactcatctctgaagaggatctgtaataacgataatcaacctctggattacaaaatttgtgaaagattgactggtattcttaactatgttgctccttttacgctatgtggatacgctgctttaatgcctttgtatcatgctattgcttcccgtatggctttcattttctcctccttgtataaatcctggttgctgtctctttatgaggagttgtggcccgttgtcaggcaacgtggcgtggtgtgcactgtgtttgctgacgcaacccccactggttggggcattgccaccacctgtcagctcctttccgggactttcgctttccccctccctattgccacggcggaactcatcgccgcctgccttgcccgctgctggacaggggctcggctgttgggcactgacaattccgtggtgttgtcggggaagctgacgtcctttccatggctgctcgcctgtgttgccacctggattctgcgcgggacgtccttctgctacgtcccttcggccctcaatccagcggaccttccttcccgcggcctgctgccggctctgcggcctcttccgcgtcttcgccttcgccctcagacgagtcggatctccctttgggccgcctccccgcatcgggaattcctagagctcgctgatcagcctcgactgtgccttctagttgccagccatctgttgtttgcccctcccccgtgccttccttgaccctggaaggtgccactcccactgtcctttcctaataaaatgaggaaattgcatcgcattgtctgagtaggtgtcattctattctggggggtggggtggggcaggacagcaagggggaggattgggaagagaatagcaggcatgctggggaccgcggcaatgctaccaaatactaattgagtgtatgtaaacttctgacccactgggaatgtgatgaaagaaataaaagctgaaatgaatcattctctctactattattctgatatttcacattcttaaaataaagtggtgatcctaactgacctaagacagggaatttttactaggattaaatgtcaggaattgtgaaaaagtgagtttaaatgtatttggctaaggtgtatgtaaacttccgacttcaactgtatagggatcctctagctagagcgtgcagctcgccgaccactaccagcagaacacccccatcggcgacggccccgtgctgctgcccgacaaccactacctgagcacccagtccgccctgagcaaagaccccaacgagaagcgcgatcacatggtcctgctggagttcgtgaccgccgccgggatcactctcggcatggacgagctgtacaagtaagtcgacc
