# Supplementary material for: Targeted delivery of a PD-1-blocking scFv by CD133-specific CAR-T cells using nonviral Sleeping Beauty transposition shows enhanced antitumour efficacy for advanced hepatocellular carcinoma
Source: BMC Med. 2023 Aug 28;21:327. doi: 10.1186/s12916-023-03016-0 (PMC10464109; doi:10.1186/s12916-023-03016-0)
Supplement: Supplementary file 2 — Additional file 2: Table S1. Correlation between CD133 expression and clinical characteristics in HCC. Table S2. Correlations between clinical characteristics and patient prognosis. Table S3. Univariate and multivariate analyses of clinical variables associated with PFS in male patients. Table S4. Univariate and multivariate analyses of clinical variables associated with OS in male patients. Figure S1. Survival analyses using different evaluation methods. Figure S2. Cell viability and CAR expression after transfection with transposon and transposase vectors at a ratio of 3:1 in T cells from PBMCs. Figure S3. Treg (CD4+CD25+Foxp3) phenotype of CD4-positive CAR T cells. Figure S4. Specific cytotoxic effects of CD133 CAR-T and PD-1s cells against CD133+ HCC cell lines. Figure S5. Enhanced specific cytotoxic effects of CD133 CAR-T and PD-1s cells among CD8- or CD4-positve CAR-T cells against CD133+ HCC cell lines. [file 12916_2023_3016_MOESM2_ESM.docx]

**Targeted delivery of a PD-1-blocking scFv by CD133-specific CAR-T cells using nonviral Sleeping Beauty transposition shows enhanced antitumor efficacy for advanced hepatocellular carcinoma**

Chaopin Yang, Jinqi You, Qiuzhong Pan, Yan Tang, Liming Cai, Yue Huang, Jiamei Gu, Yizhi Wang, Xinyi Yang, Yufei Du, Dijun Ouyang, Hao Chen, Haoran Zhong, Yongqiang Li, Jieying Yang, Yulong Han, Fengze Sun, Yuanyuan Chen, Qijing Wang, Desheng Weng, Zhongqiu Liu, Tong Xiang and Jianchuan Xia

Supplementary tables and figure legends2

Supplementary tables2

Supplementary figures and figure legends7

**Supplementary tables and figure legends**

**Supplementary tables**

**Table S1.** Correlation between CD133 expression and clinical characteristics in HCC

|  | Total  n=67(%) | CD133 expression | | *P* value |
| --- | --- | --- | --- | --- |
|  |  | **High**  **n=19**  **28.4%** | **Low**  **n=48**  **71.6%** |  |
| *Age (years)* |  |  |  |  |
| <50 | 35(52.2) | 9(47.4) | 26(54.2) | 0.616 |
| ≥50 | 32(47.8) | 10(52.6) | 22(45.8) |  |
| *Gender*  Male | 61(91.0) | 15(78.9) | 46(95.8) | 0.088 |
| Female | 6(9.0) | 4(21.1) | 2(4.2) |  |
| *HBsAg*  Negative  Positive | 3(4.5)  64(95.5) | 2(10.5)  17(89.5) | 1(2.1)  47(97.9) | 0.192 |
| *Liver cirrhosis*  No  Yes  *Tumour size*  <5 cm  ≥5 cm  *Tumour number*  Single  Multiple  *Tumour encapsulation*  No  Yes  *Histological differentiation*  Well differentiated  Moderately/Poorly differentiated  *TNM stage*  Ⅰ  Ⅱ/Ⅲ  *Portal vein invasion*  No  Yes  *Microvascular invasion*  No  Yes  *AFP*  <400 ng/ml  ≥400 ng/ml | 29(43.3)  38(56.7)  24(35.8)  43(64.2)  47(70.1)  20(29.9)  39(58.2)  28(41.8)  10(14.9)  57(85.1)  37(55.2)  30(44.8)  60(89.6)  7(10.4)  62(92.5)  5(7.5)  40(59.7)  27(40.3) | 7(36.8)  12(63.2)  8(42.1)  11(57.9)  12(63.2)  7(36.8)  14(73.7)  5(26.3)  2(10.5)  17(89.5)  9(47.4)  10(52.6)  13(68.4)  6(31.6)  19(100.0)  0(0.0)  7(36.8)  12(63.2) | 22(45.8)  26(54.2)  16(33.3)  32(66.7)  35(72.9)  13(27.1)  25(52.1)  23(47.9)  8(16.7)  40(83.3)  28(58.3)  20(41.7)  47(97.9)  1(2.1)  43(89.6)  5(10.4)  33(68.8)  15(31.3) | 0.503  0.500  0.431  0.106  0.798  0.416  **0.002**  0.344  **0.016** |

**Table S2.** Correlations between clinical characteristics and patient prognosis

| Clinical Variable | Overall survival | | Progression-free survival | |
| --- | --- | --- | --- | --- |
|  | **Hazard Ratio**  **(95% CI)** | ***P* Value** | **Hazard Ratio (95% CI)** | ***P* Value** |
| *Age*  ≥50years/<50years | 0.984(0.525,1.846) | 0.961 | 1.080(0.576,2.024) | 0.811 |
| *Gender* |  |  |  |  |
| *Male* vs. *Female* | 5.455(0.748,39.788) | 0.094 | 5.449(0.747,39.748) | 0.094 |
| *HBsAg*  Positive/Negative  *Liver cirrhosis*  Yes vs. No | 1.143(0.274,4.759)  2.159(1.092,4.267) | 0.855  **0.027** | 0.885(0.213,3.678)  2.024(1.023,4.004) | 0.866  **0.043** |
| *Tumour size*  ≥5 cm/<5 cm | 1.571(0.792,3.114) | 0.196 | 1.607(0.812,3.180) | 0.173 |
| *Tumour number*  Multiple/Single | 4.711(2.427,9.144) | **＜0.001** | 3.588(1.864,6.907) | **＜0.001** |
| *Tumour encapsulation*  Yes/No | 0.726(0.380,1.385) | 0.331 | 0.744(0.390,1.419) | 0.369 |
| *Histological differentiation*  Moderately or Poorly differentiated/Well differentiated | 0.948(0.418,2.149) | 0.898 | 1.013(0.447,2.300) | 0.974 |
| *TNM stage*  Ⅱ/Ⅲ vs. I  *Portal vein invasion*  Yes/No  *Microvascular invasion*  Yes/No  *AFP*  ≥400 ng·ml^-1^/<400 ng·ml^-1^  *CD133 expression*  High/Low | 3.383(1.755,6.521)  3.066(1.274,7.376)  0.600(0.145,2.493)  0.830(0.426,1.618)  1.024(0.509,2.059) | **＜0.001**  **0.012**  0.483  0.585  0.947 | 3.483(1.807,6.711)  3.529(1.456,8.554)  0.584(0.141,2.424)  0.773(0.397,1.505)  1.050(0.521,2.114) | **＜0.001**  **0.005**  0.459  0.449  0.892 |

**Table S3.** Univariate and multivariate analyses of clinical variables associated with PFS in male patients

| Clinical Variable | Progression-free survival | | | | |
| --- | --- | --- | --- | --- | --- |
|  | **Univariate Cox** | | | **Multivariate Cox** | |
|  | **Hazard ratio (95% CI)** | ***P* Value** | **Hazard ratio**  **(95% CI)** | | ***P* Value** |
| *Age*  ≥50 years/<50 years | 1.094(0.487,2.454) | 0.828 |  | |  |
| *HBsAg*  Positive/Negative  *Liver cirrhosis*  Yes vs. No | 0.411(0.053,3.213)  1.209(0.477,3.063) | 0.397  0.689 |  | |  |
| *Tumour size*  ≥5 cm/<5 cm | 1.938(0.750,5.009) | 0.172 |  | |  |
| *Tumour number*  Multiple/Single | 1.815(0.706,4.666) | 0.216 |  | |  |
| *Tumour encapsulation*  Yes/No | 1.140(0.448,2.901) | 0.783 |  | |  |
| *Histological differentiation*  Moderately or Poorly Differentiated/Well Differentiated | 3.180(0.426,23.744) | 0.259 |  | |  |
| *TNM stage*  Ⅲ/Ⅱ  *Portal vein invasion*  Yes/No  *Microvascular invasion*  Yes/No  *AFP*  ≥400 ng·ml^-1^/<400 ng·ml^-1^  *CD133 expression*  High/Low | 3.863(1.285,11.610)  3.690(1.337,10.186)  0.201(0.045,0.893)  0.998(0.434,2.296)  3.378(1.247,9.152) | **0.016**  **0.012**  **0.035**  0.996  **0.017** | 3.270(1.049,10.192)  2.600(0.926,7.301) | | **0.041**  0.070 |

**Table S4.** Univariate and multivariate analyses of clinical variables associated with OS in male patients

| Clinical Variable | Overall survival | | | | |
| --- | --- | --- | --- | --- | --- |
|  | **Univariate analysis** | | | **Multivariate analysis** | |
|  | **Hazard Ratio (95% CI)** | ***P* Value** | | **Hazard Ratio (95% CI)** | ***P* Value** |
| *Age*  ≥50years/<50years | 0.816(0.362,1.840) | 0.624 |  | |  |
| *HBsAg*  Positive/Negative  *Liver cirrhosis*  Yes vs. No | 1.725(0.229,12.996)  1.391(0.550,3.519) | 0.597  0.486 |  | |  |
| *Tumour size*  ≥5 cm/<5 cm | 1.495(0.547,4.090) | 0.433 |  | |  |
| *Tumour number*  Multiple/Single | 3.747(1.366,10.283) | **0.010** | 3.500(1.274,9.619) | | **0.015** |
| *Tumour encapsulation*  Yes/No | 1.212(0.472,3.114) | 0.690 |  | |  |
| *Histological differentiation*  Moderately or Poorly Differentiated/Well Differentiated | 2.587(0.342,19.561) | 0.357 |  | |  |
| *TNM stage*  Ⅲ/Ⅱ  *Portal vein invasion*  Yes/No  *Microvascular invasion*  Yes/No  *AFP*  ≥400 ng·ml^-1^/<400 ng·ml^-1^  *CD133 expression*  High/Low | 2.968(0.973,9.054)  2.712(1.032,7.124)  0.210(0.048,0.928)  1.189(0.518,2.733)  2.932(1.167,7.367) | 0.056  **0.043**  **0.039**  0.683  **0.022** | 2.609(1.039,6.546) | | **0.041** |


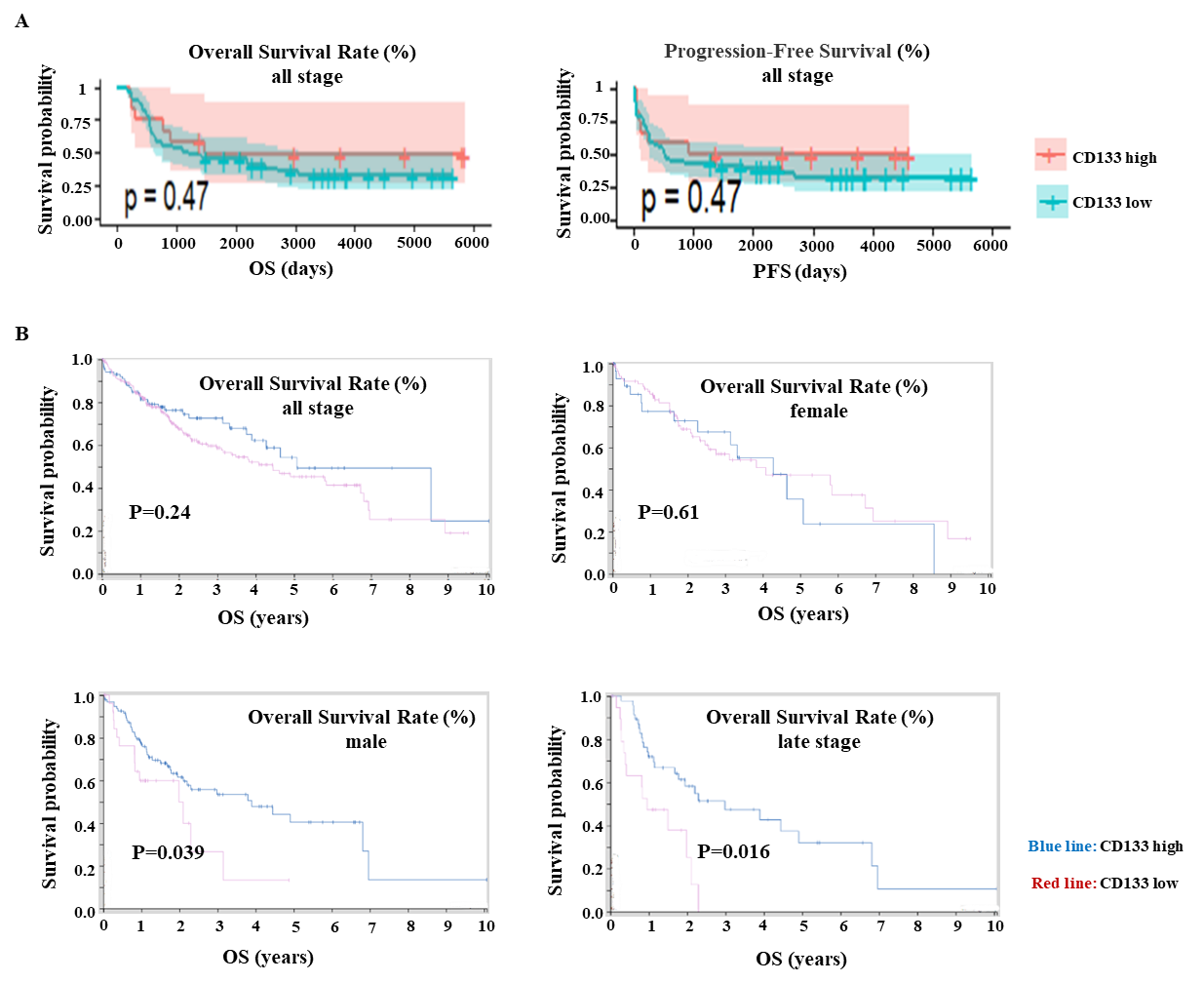
**Supplementary figures and figure legends**

**Figure S1.** Survival analyses using different evaluation methods. **A** Kaplan–Meier curve depicting survival analyses of 67 hepatocellular carcinoma patients. The *P* value was calculated by the log-rank test. **B** Survival analyses of patients at different stages from The Human Protein Atlas.

**
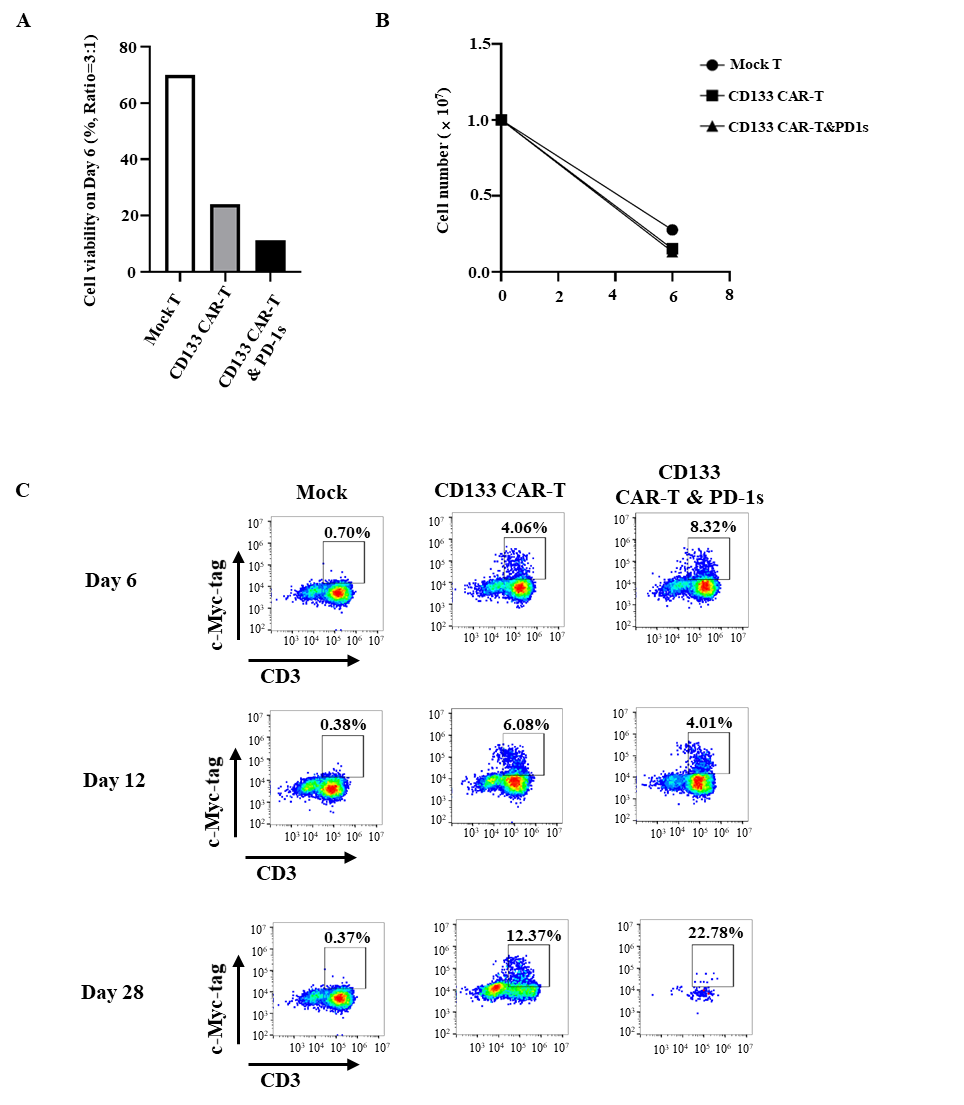
Figure S2.** Cell viability and CAR expression after transfection with transposon and transposase vectors at a ratio of 3:1 in T cells from PBMCs.

**A** The cell viability at a ratio of 3:1 6 days after electrotransfection was assessed by 7-AAD staining. **B** The cell number decreased to nearly 1/3. **C** Flow cytometry analysis of CAR expression using c-Myc-tag expression as a surrogate on days 6, 12, and28.


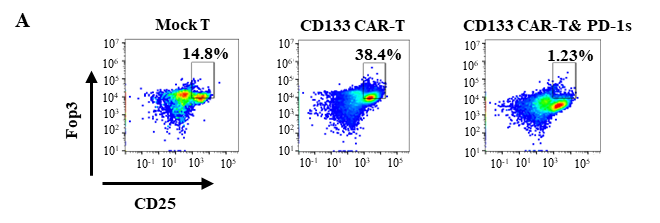


**Figure S3.** Treg (CD4+CD25+Foxp3) phenotype of CD4-positive CAR T cells.

**
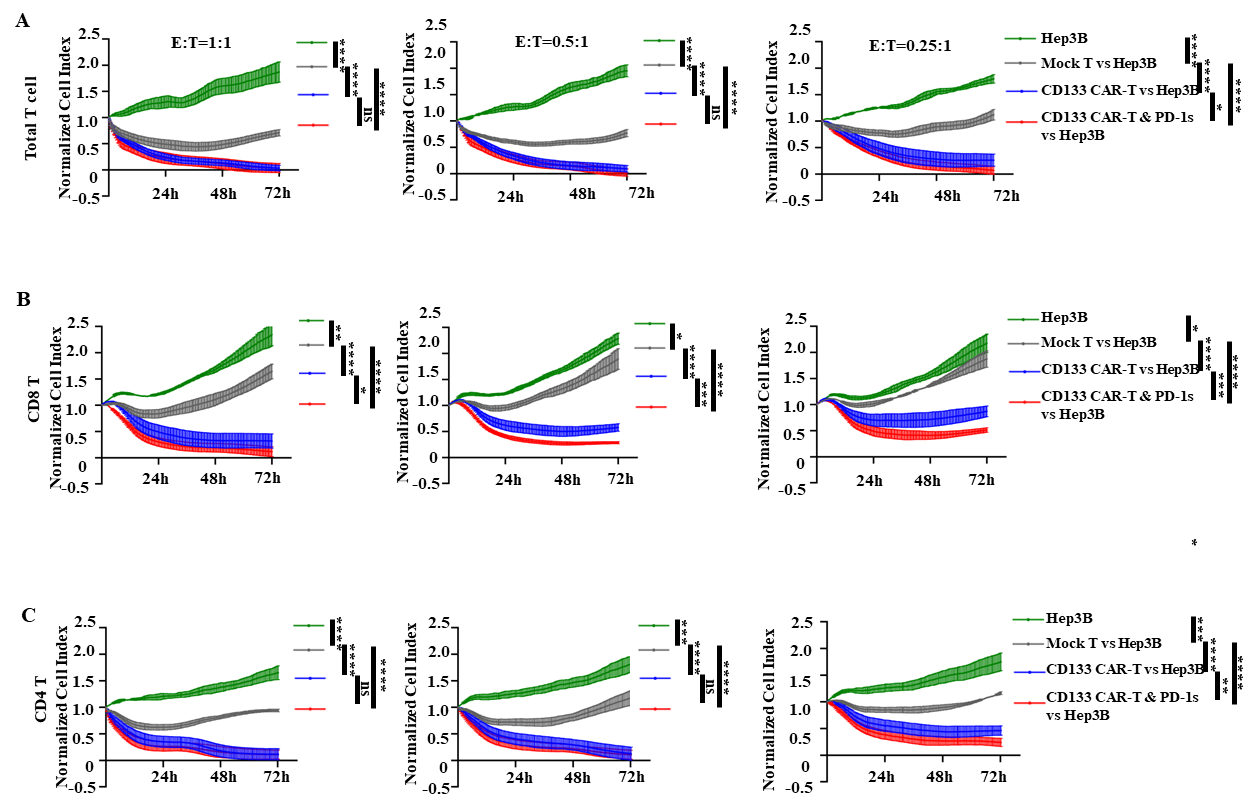
****Figure S4.** Specific cytotoxic effects of CD133 CAR-T and PD-1s cells against CD133+ HCC cell lines. **A** Continuous graphical output of cell index values determined using the xCELLigence Impedance system from medium, NT, Mock T, CD133 CAR-T and CD133 CAR-T & PD-1s for 72h. **B** Continuous graphical output of cell index values determined using the xCELLigence Impedance system from medium, CD8-positive Mock T, CD8-positive CD133 CAR-T and CD8-positive CD133 CAR-T & PD-1s for 72h. **C** Continuous graphical output of cell index values determined using the xCELLigence Impedance system from medium, CD4-positive Mock T, CD4-positive CD133 CAR-T and CD4-positive CD133 CAR-T & PD-1s for 72h. Mean ± SD, n = 4, two-tailed unpaired Student’s *t test*, **P* < 0.05, ***P* < 0.01, ****P* < 0.001, *****P* < 0.0001, ns, not significant. The total number of T cells, CD8 T cells, CD4 T cells were different due to the differences in CAR positivity among total T cells, CD8 T cells and CD4 T cells.

**
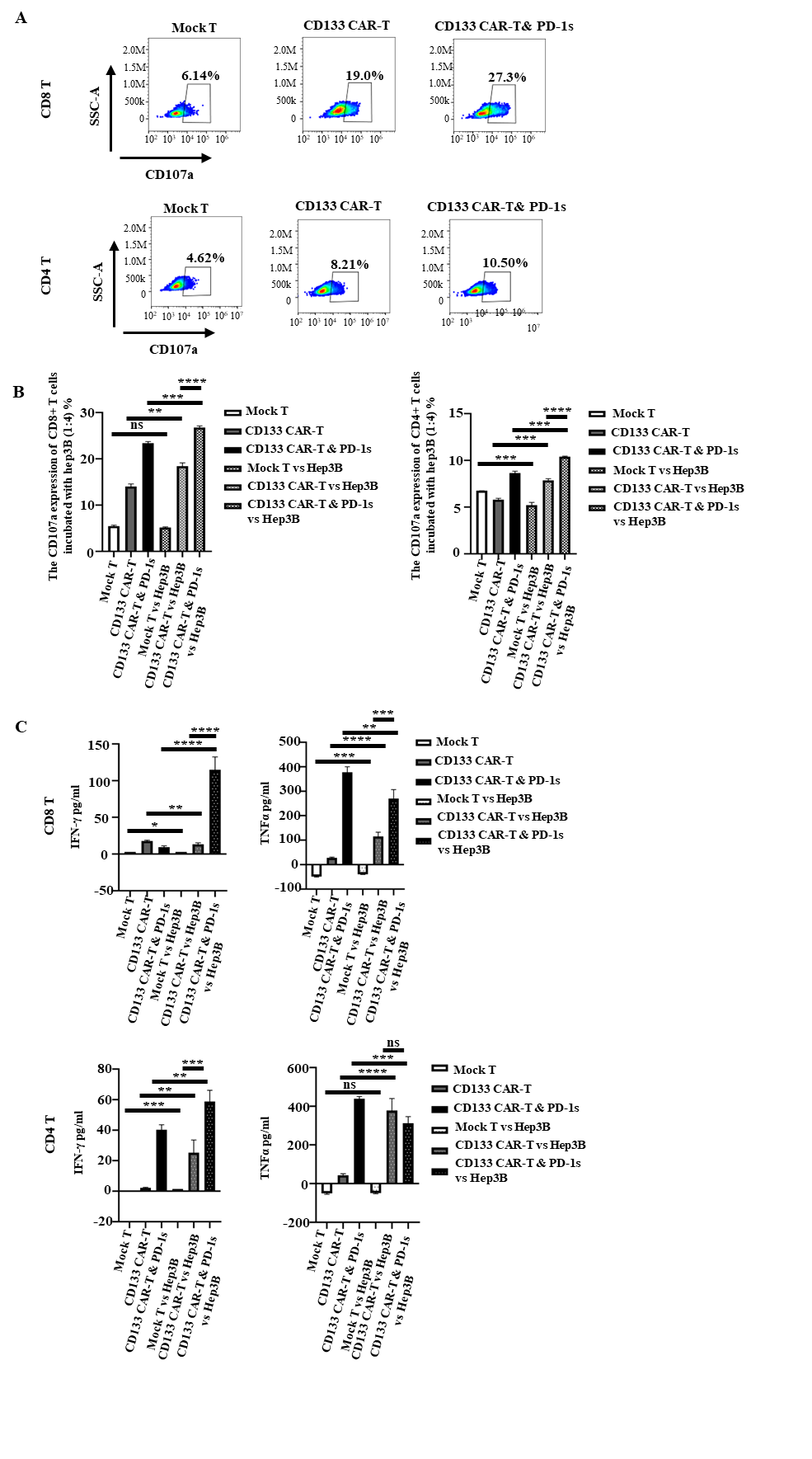
**

**Figure S5.** Enhanced specific cytotoxic effects of CD133 CAR-T & PD-1s cells among CD8- or CD4-positve CAR-T cells against CD133+ HCC cell lines.

**A** Representative flow cytometric image of CD107a expression in Mock T cells, CD133 CAR-T cells, and CD133 CAR-T & PD-1s cells incubated with tumour cells for 24 hours. **B** The frequency of CD107a expression on Mock T, CD133 CAR-T, and CD133 CAR-T & PD-1s cells alone or incubated with Hep3B cells was determined by flow cytometric analysis. **C** The Cytokine production (IFNγ and TNFα) by Mock T cells, CD133 CAR-T cells, and CD133 CAR-T & PD-1s cells incubated or not incubated with tumour cells for 24 hours was measured by ELISA. Mean ± SD, n = 4, two-tailed unpaired Student’s *t test*, **P* < 0.05, ***P* < 0.01, ****P* < 0.001, *****P* < 0.0001, ns, not significant.
