# Supplementary figures and images for: Targeted delivery of a PD-1-blocking scFv by CD133-specific CAR-T cells using nonviral Sleeping Beauty transposition shows enhanced antitumour efficacy for advanced hepatocellular carcinoma
Source: BMC Med. 2023 Aug 28;21:327. doi: 10.1186/s12916-023-03016-0 (PMC10464109; doi:10.1186/s12916-023-03016-0)

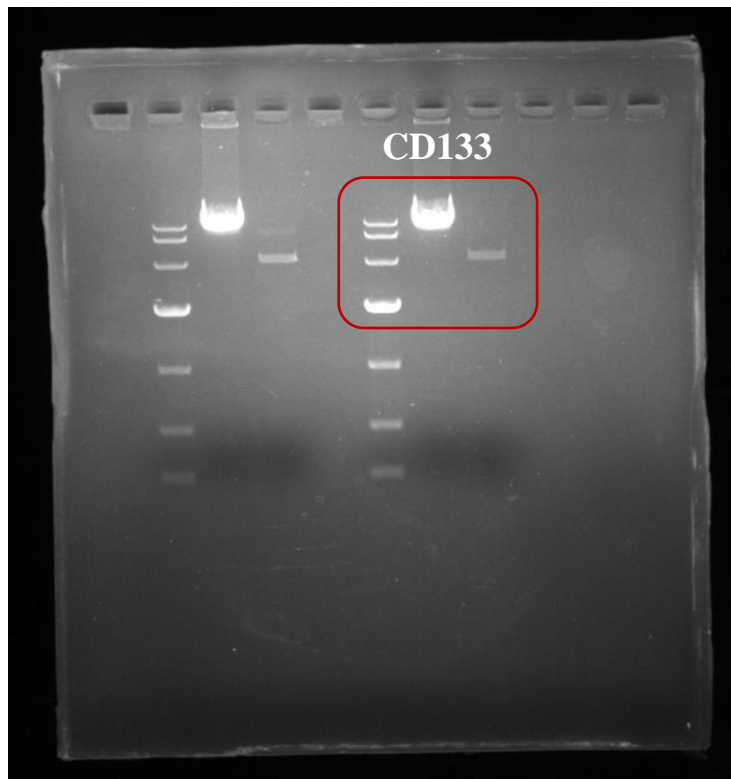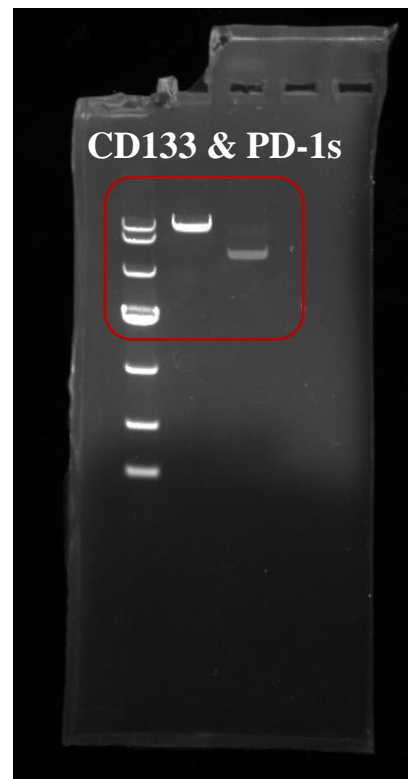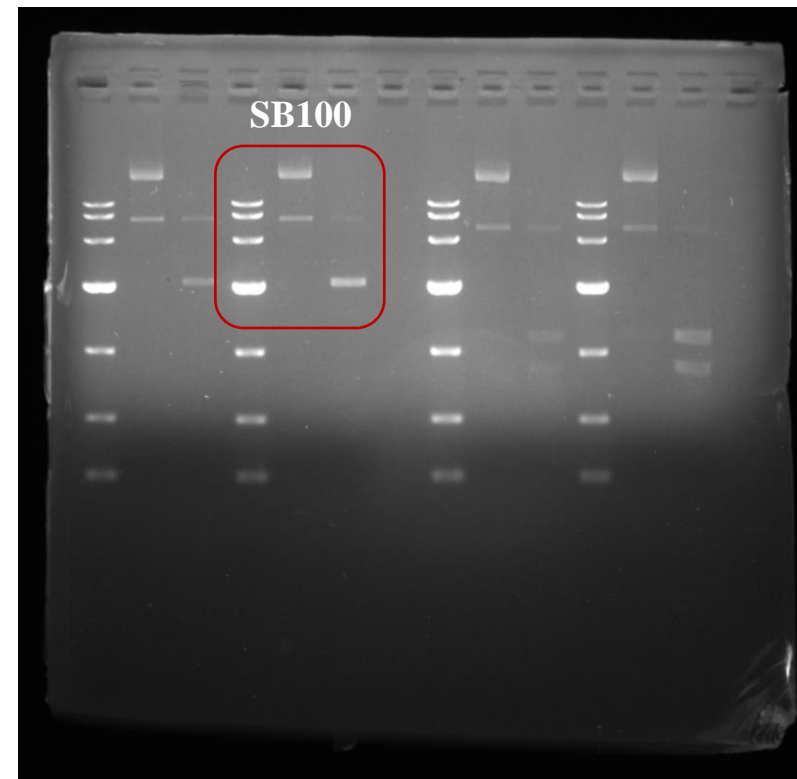

**Figure 2B**

**GAPDH**

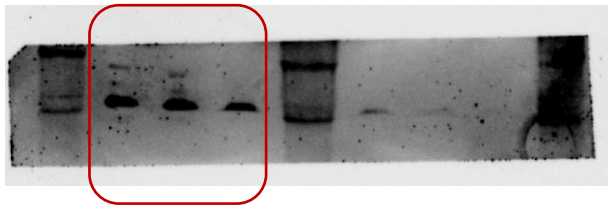

**PD-1s**

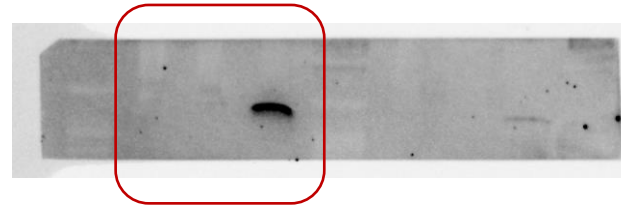

**Figure 2F**

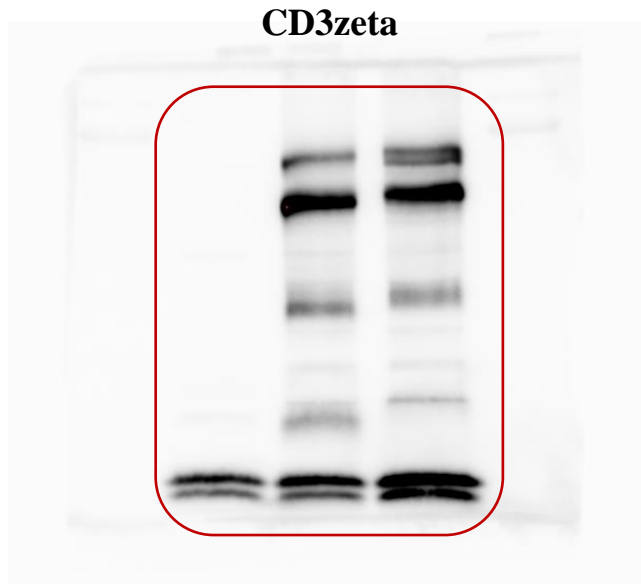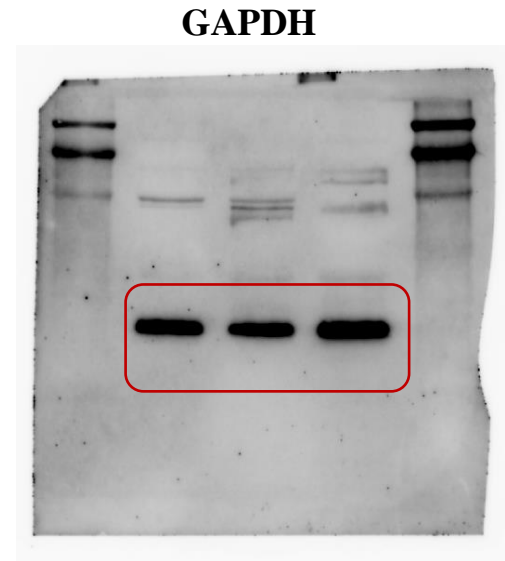

**Figure 3B**

Supplement: Supplementary file 3 — Additional file 3. Images of the original, uncropped gels/blots in Fig. 2 B, F and Fig. 3B. [file 12916_2023_3016_MOESM3_ESM.pdf]
